# Antiviral Susceptibility of Clade 2.3.4.4b H5N1 and H5N5 Influenza Viruses Detected in Birds in Russia During 2021–2022

**DOI:** 10.3390/v18101126

**Published:** 2026-10-10

**Authors:** Svetlana V. Svyatchenko, Natalia I. Goncharova, Ivan M. Susloparov, Natalia N. Vasiltsova, Natalia P. Kolosova, Marina L. Egorova, Tatyana V. Tregubchak, Natalia V. Danilchenko, Tatyana N. Ilyicheva, Vasiliy Y. Marchenko

**Affiliations:** State Research Center of Virology and Biotechnology “Vector” Rospotrebnadzor, Koltsovo 630559, Russia; goncharova_ni@vector.nsc.ru (N.I.G.); imsous@vector.nsc.ru (I.M.S.); vasiltsova_nn@vector.nsc.ru (N.N.V.); egorova_ml@vector.nsc.ru (M.L.E.); tregubchak_tv@vector.nsc.ru (T.V.T.); danilchenko_nv@vector.nsc.ru (N.V.D.); ilicheva_tn@vector.nsc.ru (T.N.I.); marchenko_vyu@vector.nsc.ru (V.Y.M.)

**Keywords:** influenza, H5N1, H5N5, 2.3.4.4b, antiviral susceptibility, neuraminidase inhibitors, NA-Y253H, oseltamivir, IC_50_

## Abstract

In this study, we aimed to analyze the antiviral susceptibility of clade 2.3.4.4b H5N1 and H5N5 influenza viruses detected in Russia during 2021–2022. During this period, we investigated 40 avian influenza outbreaks in wild and domestic birds, isolating 109 H5N1 and 9 H5N5 strains. Using a genetic approach, we analyzed the presence of markers associated with reduced susceptibility to baloxavir and M2 blockers, as well as markers of reduced inhibition by neuraminidase inhibitors (NAIs). Additionally, the isolated strains were tested in a functional assay to evaluate the inhibitory activity of oseltamivir, zanamivir, and peramivir against viral neuraminidase. None of the isolates harbored genetic markers of reduced susceptibility to M2 blockers and baloxavir or reduced inhibition by NAIs. Functional assay showed that the median IC_50_ values of oseltamivir, zanamivir, and peramivir were 1.73, 0.49, and 0.16 nM for the H5N1 subtype, and 0.65, 1.13, and 0.12 nM for the H5N5 subtype, respectively. None of the tested strains demonstrated a ≥10-fold increase in NAI IC_50_ values compared to the corresponding subtype medians, and were thus classified as having a normal level of inhibition. The median IC_50_ values of the NAIs determined for the tested avian viruses were comparable to those of a susceptible seasonal A(H1N1)pdm09 strain, except for a slight reduction in oseltamivir activity against H5N1 viruses (4.3-fold) and zanamivir activity against H5N5 viruses (3.4-fold). Our study revealed a group of H5N1 viruses whose NA belonged to the ea5.1 genetic subclade, characterized by NA-Y253H and T452I amino acid substitutions, which exhibited a 28.8-fold decrease in oseltamivir IC_50_ values compared to the subtype median. These strains were detected in the European and Ural regions of Russia between May 2022 and January 2023, with no subsequent detections in the available surveillance data. To the best of our knowledge, such strains have not been identified outside Russia.

## 1. Introduction

Highly pathogenic avian influenza (HPAI) A(H5N1) viruses (*Alphainfluenzavirus influenzae*) of clade 2.3.4.4b are currently one of the major causative agents of influenza outbreaks in wild and domestic birds worldwide [1]. The first representatives of clade 2.3.4.4b H5N1 viruses detected in the Netherlands in 2020 seemingly emerged through reassortment between H5N8 viruses circulating at that time in the region and Eurasian low-pathogenic strains [2]. Since then, these viruses have spread to different regions of the world, continuing their evolution and undergoing additional reassortment events with local influenza strains, thereby giving rise to a multitude of different genotypes [3,4,5].

In addition to their circulation in avian hosts, clade 2.3.4.4b H5N1 influenza viruses have shown the capacity to infect a wide range of mammal species, including dairy cows [6,7]. Their limited transmission between mammals was registered or suspected on several occasions [6]. Furthermore, these viruses have occasionally demonstrated the ability to transmit from infected animals to humans in occupational settings or on backyard farms. To date, more than 80 cases of human infection with clade 2.3.4.4b H5N1 influenza viruses have been reported [8]. Most of them were mild, presenting with conjunctivitis, self-limiting respiratory symptoms, or both [9], although several severe [10] and even fatal cases [11,12] have occurred. The overall global public health risk is considered low at the moment [13]. However, given the ability of influenza viruses to rapidly evolve, thorough monitoring and the preliminary development of mitigation strategies remain of utmost importance for pandemic preparedness [14].

Antiviral drug prophylaxis and therapy play a major role in pandemic management, along with vaccination and non-pharmaceutical public health measures [15,16,17]. To date, there are three major groups of antiviral drugs approved for influenza management, including neuraminidase inhibitors (NAIs), cap-dependent endonuclease inhibitors (including baloxavir), and M2 blockers. Even though data on their clinical effectiveness for zoonotic influenza prophylaxis and treatment are limited [18], experimental data suggest that they could possibly become a powerful tool for influenza H5N1 management in the event of a pandemic [19,20]. This underlines the importance of antiviral susceptibility surveillance among circulating HPAI clade 2.3.4.4b H5N1 viruses. Because antiviral susceptibility may be influenced by substitutions not previously characterized as resistance markers, phenotypic and functional testing remain an important complement to genetic surveillance. At present, the majority of published data on the antiviral susceptibility of clade 2.3.4.4b H5N1 viruses focus on strains detected in North America [21,22,23], while data on Eurasian strains remain sparse.

In this study, we aimed to evaluate the antiviral susceptibility of clade 2.3.4.4b H5N1 influenza viruses detected in Russia in 2021–2022 during the first two years of their circulation in the country. Specifically, we performed a genetic analysis of the segments encoding viral proteins targeted by approved anti-influenza drugs to detect previously described genetic markers of reduced susceptibility. Additionally, we assessed the potency of oseltamivir, zanamivir, and peramivir to inhibit the neuraminidase activity of isolated clade 2.3.4.4b H5N1 strains, in comparison with H5N5 viruses occasionally detected in Russia in 2021 and representative seasonal influenza strains.

## 2. Materials and Methods

### 2.1. Sampling, RT-PCR Testing and Virus Isolation

Sampling during avian influenza outbreaks was conducted by local veterinary services. Specimens collected from birds (cloacal swabs or organ fragments) were tested for H5N1 influenza virus using reverse transcription polymerase chain reaction (RT-PCR) at the regional Hygienic and Epidemiological Centers of Rospotrebnadzor. Samples positive for influenza H5N1 were sent to the biosafety level 3 (BSL-3) laboratory of the State Research Center of Virology and Biotechnology “Vector”, Rospotrebnadzor (SRC VB “Vector”) for virus isolation in embryonated chicken eggs (ECEs). Ten-day-old ECEs were inoculated with 100 µL of the specimens into the allantoic cavity, followed by a 2-day incubation at 37 °C. Eggs were then chilled at −20 °C for 30 min, and the allantoic fluid was harvested. Virus isolation was confirmed using a hemagglutination reaction and an RT-PCR assay [24].

### 2.2. Sequencing and Genetic Analysis

Sequencing was performed on both original samples and ECE isolates whenever the clinical specimen contained sufficient RNA load. For H5N1 viruses, sequences were determined for both types of material in 39 cases, for the ECE isolate only—in 69 cases, and for the original sample only—in one case. For H5N5 viruses, sequencing was performed for both types of material in one case, while for the other eight strains, only ECE isolates were sequenced. Sequencing was performed on an Illumina MiSeq platform (Illumina, Inc., San Diego, CA, USA) as previously described [25]. The determined nucleotide sequences were deposited in the Global initiative on sharing all influenza data (GISAID) database [26].

Sequence alignment was performed using MAFFT v7.508 [27]. Amino acid substitutions were analyzed using MEGA 6 software [28]. Maximum likelihood phylogenetic analysis was performed in IQ-TREE v2.0.7 [29] applying the best-fitted nucleotide substitution model selected by ModelFinder [30] with 1000 bootstrap replicates. Trees were visualized using the ggtree R package v4.2.0 [31]. Amino acid substitutions were mapped onto the phylogenetic tree using Treesub software [32].

To assess the prevalence of an NA-Y253H amino acid substitution in circulating clade 2.3.4.4b H5N1 influenza viruses, available neuraminidase sequences from the GISAID database were aligned using MAFFT v7.508 [27], then manually curated and deduplicated using SeqKit v2.13.0 [33].

A search for the most closely related sequences was performed using the Basic Local Alignment Search Tool (BLAST) v2.17.0 [34] in the National Center for Biotechnology Information (NCBI) [34] and GISAID [26] databases.

### 2.3. Fluorescence Neuraminidase Inhibition Assay

The potency of oseltamivir (TRC (Toronto Research Chemicals), Toronto, ON, Canada), zanamivir (Sigma-Aldrich, St. Louis, MO, USA), and peramivir (TRC, Toronto, ON, Canada) in inhibiting neuraminidase activity was studied in a fluorescence neuraminidase inhibition assay (NIA) for all isolated H5Nx and control seasonal influenza viruses using the 2′-(4-methylumbelliferyl)-α-D-N-acetylneuraminic acid (MUNANA) substrate (Biosynth, Staad, Switzerland) according to [35]. Half-maximum inhibitory concentrations (IC_50_) were calculated based on the obtained data for all isolates and drugs using Origin(Pro), Version 2019b (OriginLab Corporation, Northampton, MA, USA).

Median IC_50_ values (MedIC_50_) of the tested drugs were calculated for groups of viruses belonging to various subtypes and genetic groups to assess their baseline susceptibility levels. Comparison of the data obtained for various subtypes and genetic groups was performed using the non-parametric Kruskal–Wallis test with post hoc Dunn’s test in Origin.

Box-and-whisker plot analysis of IC_50_ values was performed according to the Tukey method in Origin. Upper outliers were defined as data points exceeding the 75th percentile plus 1.5 times the interquartile range (IQR, the difference between the 75th and 25th percentiles). Lower outliers were defined as data points below the 25th percentile minus 1.5 times the IQR.

Interpretation of the obtained data was performed using criteria established by the World Health Organization (WHO) expert working group on surveillance of influenza antiviral susceptibility (Antiviral Working Group, AVWG) [36]. The fold-change was calculated as the ratio of the IC_50_ value determined for an individual tested strain to the MedIC_50_ value determined for all tested strains of the same subtype and/or NA genetic group. Inhibition of a tested strain was considered normal (NI—normal inhibition) if the determined fold-change was below 10. Inhibition was considered reduced (RI—reduced inhibition) or highly reduced (HRI—highly reduced inhibition) if the fold-change was 10–100 and >100, respectively [37].

Reference seasonal influenza A(H1N1)pdm09 viruses A/California/07/2009 and A/North Carolina/39/2009 used as assay controls were kindly provided by the WHO Collaborating Center for Surveillance, Epidemiology and Control of Influenza, Influenza Division, Centers for Disease Control and Prevention, Atlanta, USA, in 2017. The reference seasonal influenza B/Victoria strain B/Austria/1359417/2021 was kindly provided by the WHO Collaborating Centre for Reference and Research on Influenza, Worldwide Influenza Centre, The Francis Crick Institute, London, in 2021.

## 3. Results

### 3.1. Virus Isolation

Clade 2.3.4.4b H5N1 influenza viruses were first detected in Russia in the summer of 2021. Throughout 2021 and 2022, SRC VB “Vector” received H5N1-positive avian samples from 40 outbreaks involving wild and domestic birds across four geographic territories in Russia (European Russia, Ural, Siberia, Russian Far East) (Figure 1).

In total, 109 H5N1 strains were isolated from received samples in ECEs (Table S1). Nucleotide sequences of HA, NA, PA, and MP segments were determined for all isolated strains. Analysis of HA sequences confirmed that they belonged to the 2.3.4.4b clade.

Additionally, we studied nine influenza H5N5 strains of the 2.3.4.4b clade that were isolated during two outbreaks in wild birds in European Russia (Astrakhan region and Republic of Kalmykia) in 2021 (Table S1).

### 3.2. Genetic Analysis of NA Segments of H5N1 Influenza Viruses

To assess the genetic diversity of neuraminidase (NA) segments among the studied clade 2.3.4.4b H5N1 viruses and its potential impact on inhibition by NAIs, we carried out a phylogenetic analysis (Figure 2). The phylogenetic tree inferred for NA segments showed that an overwhelming majority of the studied Russian strains belonged to a large monophyletic group designated here as X1. According to the ggFlu global H5 genotyping system [38], this NA cluster is designated as “Eurasian_GP2_N1 (NA_155)”. It likely originated from a low-pathogenic Eurasian A(HxN1) virus [2] and represented the predominant NA variant among European 2.3.4.4b H5N1 viruses in 2020–2022 [5].

Only one studied Russian outbreak was associated with viruses whose NA clustered separately (isolates A/chicken/Russia/56-1V/2022, A/chicken/Russia/56-2V/2022, A/chicken/Russia/56-4V/2022, A/chicken/Russia/56-5V/2022). This NA genetic group is designated here as X2, corresponding to “Eurasian_GP2_N1 (NA_159)” cluster in the ggFlu global H5 genotyping system [38]. A search of publicly available sequence databases revealed only one other clade 2.3.4.4b H5N1 virus bearing an X2-neuraminidase globally (A/garganey/Egypt/SJCEIRR-RA20851OP/2022). According to BLAST analysis, the X2 neuraminidase segment was most closely related to low pathogenic avian influenza (LPAI) A(H1N1) viruses detected in Siberia (A/Shoveler/Chany_Lake/62/2019), Eastern Europe (A/Fieldfare/Bogatyr-Ukraine/M218914/86-90/24-02/2021), and Egypt (A/northern pintail duck/Egypt/MB-D-2211C/2019), as well as non-goose/Guangdong lineage H5N1 viruses detected in Italy in 2021 (Table S2, Figure 2). Phylogenetic analysis suggested that NA segments of the X1 and X2 groups originated independently from closely related Eurasian LPAI A(HxN1) viruses, although, as previously noted [2], limited surveillance data constrain definitive conclusions. Viruses of the X2 NA genetic group harbored 13–21 amino acid substitutions (AASs) in NA compared to the X1 strains analyzed in this study (Figure 2), corresponding to 95.52–97.23% NA amino acid sequence identity. Within the X1 group, amino acid sequence identity of NA ranged from 97.02 to 100% (0–14 AASs).

Within the X1 NA genetic group, the analyzed Russian viruses clustered into two subgroups, which were classified as ea3 and ea5 according to the GenoFlu nomenclature [39] (Figure 2). During the first year of virus circulation in Russia, outbreaks were predominantly registered in the European and Ural regions. The majority of them were associated with viruses bearing an NA segment of the ea5 subgroup, while viruses with an ea3 NA segment were registered less frequently. In 2022, there was a difference in the geographic distribution of clade 2.3.4.4b H5N1 viruses belonging to different NA genetic subgroups. Specifically, viruses with an ea5 NA were registered in European Russia, while viruses with an ea3 NA were detected in the Russian Far East (Figure 3).

Within the ea3 NA clade, Russian viruses were distributed between two subclades, designated in this article as ea3.1 and ea3.2. Viruses of the ea3.1 NA genetic subclade were registered in the Ural region in 2021 and later in the Far Eastern region in 2022. Viruses of this NA subclade harbored characteristic AASs NA-Y44H, S70N, Q308H/R, V394I, and A395E. Viruses of the ea3.2 NA subclade were registered on one occasion in European Russia in 2021 and subsequently during several outbreaks in the Russian Far East in 2022. Viruses of this subclade typically carried AASs NA-S70N and W399L (Figure 2 and Figure 3). (Hereinafter, neuraminidase amino acid numbering for H5N1 viruses is given according to the reference 2.3.4.4b H5N1 strain A/bald eagle/Florida/W22-134-OP/2022 [37] with full-length neuraminidase without deletions, unless specified otherwise).

Of the ten outbreaks associated with ea5 viruses registered in 2022 in the European and Ural regions, seven were caused by viruses whose NA belonged to a subclade that emerged within the ea5 NA group and was designated as “ea5.1” in this study. These viruses carried distinctive AASs NA-Y253H and T452I (Figure 2 and Figure 3).

Comparison of the sequencing results obtained for the original samples and their corresponding ECE isolates showed that for all 39 H5N1 strains with available data (including 10 strains of subclade ea3.2, 17 strains of clade ea5, and 12 strains of subclade ea5.1), the neuraminidase amino acid sequences were identical between both types of material, confirming that cultivation in ECEs did not introduce any additional AASs.

The neuraminidase amino acid sequences of the studied viruses were analyzed for the presence of AASs previously associated with altered NA inhibition by anti-neuraminidase drugs in H5N1 viruses, according to the list of genetic markers recommended for monitoring by WHO experts [37]. The analyzed strains carried no specific NA AASs associated with reduced or highly reduced inhibition specified in the list; however, a subset of strains harbored alternative amino acid residues at positions mentioned in it (NA-V116I in 9 strains (*N* = 9), NA-I117M (*N* = 3), NA-Q136P (*N* = 2), NA-V149D (*N* = 1), NA-K150N (*N* = 1), and NA-K150T (*N* = 1)) (Table S3).

### 3.3. Genetic Analysis of NA Segments of H5N5 Influenza Viruses

Phylogenetic analysis showed that both studied H5N5 outbreaks were associated with viruses whose NA segments belonged to the ea6 cluster according to the GenoFlu nomenclature (Figure 4) [39]. Compared to the Eurasian LPAI H12N5 strains from which their NA segment was seemingly derived, viruses of the ea6-group harbored a specific subset of AASs in NA (L23I, K169R, D261G, S357N, and V392I). While viruses from the Astrakhan outbreak had a full-length NA stalk, the isolate from Kalmykia carried a 22-codon deletion spanning positions 47 to 68 in the NA segment. This strain belonged to a distinct subclade comprising analogous strains with this deletion and AASs NA-I113T, S417G, and R435K (Figure 4). (Hereinafter, neuraminidase amino acid numbering for H5N5 viruses is given according to the reference H6N5 strain A/aquatic bird/Korea/W69/2005 [37] with full-length neuraminidase without deletions, unless specified otherwise).

The genetic analysis showed that none of the studied H5N5 viruses had previously described genetic markers of reduced inhibition by NAIs established for the N5 neuraminidase [37].

### 3.4. Inhibition of Neuraminidase Activity by Oseltamivir, Zanamivir, and Peramivir

All isolated strains were tested in a fluorescence neuraminidase inhibition assay. The median IC_50_ (MedIC_50_) value of oseltamivir determined for the H5N1 subtype stood at 1.73 nM. To assess possible differences in oseltamivir potency between viruses from different NA genetic groups, MedIC_50_ values were determined separately for each group (Figure 5A, Tables S4 and S5). Median IC_50_ values of the ea3.1, ea3.2, ea5, and X2 groups differed by no more than 1.2-fold from the overall H5N1 subtype median. Viruses of the ea5.1 group with characteristic AASs NA-Y253H and T452I differed greatly from all other groups; their MedIC_50_ was 28.8-fold lower than the overall subtype median.

Box-and-whisker plot analysis of the oseltamivir data revealed two lower outliers in the ea5 group (strains A/goose/Chelyabinsk/34-1V/2021 (NA-S442I) and A/goose/Tyumen/359-12/2021 (NA- E47G, V149D, and I436V)), showing a 3.4- and 1.7-fold IC_50_ decrease compared to the ea5-group median. One upper outlier was registered in the ea3.2 group, exhibiting a 1.5-fold increase in the oseltamivir IC_50_ value relative to the ea3.2-group median (A/chicken/Magadan/14-1V/2022 (NA- G41E, Q43R, T55A, A79T, and V116I)). Other H5N1 genetic subgroups did not present any outliers (Figure 5A, Table S6).

The median oseltamivir IC_50_ determined for H5N5 viruses was 0.65 nM. There was one upper outlier in the H5N5 sample (A/dalmatian pelican/Astrakhan/344-12/2021 (NA-D303N and I428V)) with an IC_50_ exceeding the subtype median by a factor of 1.8 (Figure 5A, Tables S4 and S6).

The Median IC_50_ of zanamivir determined for all tested H5N1 viruses was 0.49 nM. Viruses of the ea3.2 group had a zanamivir MedIC_50_ approximately two-fold higher than strains from the ea3.1 and ea5 groups. The ea3.2 group had one upper outlier (A/chicken/Magadan/14-1V/2022 (NA-G41E, Q43R, T55A, A79T, and V116I)) whose IC_50_ value exceeded the ea3.2 median by 1.7-fold. The ea5 group had six upper outliers, whose IC_50_ values were within a factor of 1.6 of the ea5 median. Compared to the ea5 group, IC_50_ values of the ea5.1 strains were slightly increased, with a median fold-difference of 1.6. Viruses of the X2 subgroup had IC_50_ values comparable to the X1 subgroup median (1.2-fold change of MedIC_50_, *p* > 0.05) (Figure 5B, Tables S4–S6).

The median zanamivir IC_50_ determined for H5N5 viruses was 1.13 nM. A single upper outlier was detected among the H5N5 viruses (A/dalmatian pelican/Astrakhan/344-12/2021 (NA-D303N and I428V)), with its IC_50_ exceeding the subtype median by a factor of 3.9 (Figure 5B, Table S6).

The median peramivir IC_50_ determined for all H5N1 viruses was 0.16 nM. On average, viruses of the ea5.1 group had 1.2-fold decreased IC_50_ values compared to the ea5 group. There were two upper outliers in the ea5 group whose IC_50_ values exceeded the ea5 median by a factor of 1.4–1.8 (A/goose/Chelyabinsk/34-1V/2021 (NA-S442I) and A/goose/Tyumen/359-12/2021 (NA- E47G, V149D, and I436V)). In the ea5.1 group, box-and-whisker plot analysis revealed one upper and one lower outlier whose IC_50_ values were, nonetheless, very close to the ea5.1 median. Viruses of the X2 subgroup exhibited IC_50_ values comparable to the X1 subgroup median (Figure 5C, Tables S4–S6).

The median peramivir IC_50_ determined for H5N5 viruses was 0.12 nM. There were no outliers in the H5N5 group (Figure 5C).

A comparison of tested avian viruses with seasonal strains included as controls in the assay showed that H5N1 influenza viruses on average exhibited 4.3-fold higher oseltamivir IC_50_ values compared to a susceptible seasonal A(H1N1)pdm09 virus, while zanamivir and peramivir IC_50_ values were comparable (~1.4-fold difference). Comparison of H5N5 viruses with a susceptible A(H1N1)pdm09 strain showed that zanamivir IC_50_ values were on average 3.4-fold higher for H5N5 strains, while oseltamivir and peramivir IC_50_ values were comparable (≤1.6-fold difference) (Table S4).

In comparison with the IC_50_ values of a susceptible seasonal B/Victoria virus, MedIC_50_ values of H5N1 and H5N5 viruses were 18.2- and 48.3-fold lower for oseltamivir, 13.6- and 5.8-fold lower for zanamivir, and 26.2- and 35.3-fold lower for peramivir (Table S4).

### 3.5. Screening for Genotypic Markers of Reduced Susceptibility to M2 Blockers and PA Inhibitors

Analysis of M2 protein sequences showed that none of the 109 studied H5N1 viruses had genotypic markers of reduced susceptibility to M2 blockers, such as M2-L26F, V27A/T, A30S/V/T, S31N, and G34E. Their detection frequency was also low in other parts of the world, ranging from 0.3–1.4% among clade 2.3.4.4b H5N1 viruses detected in birds and mammals in the US in 2022 and 2024–2025, respectively [21,22], to 3.3% among H5N1 viruses detected globally in 2022–2023 [40].

The nine isolated H5N5 viruses also did not carry genetic markers of reduced susceptibility to M2 blockers.

Analysis of PA segments showed that none of the studied H5N1 and H5N5 influenza viruses harbored genetic markers associated with reduced susceptibility to PA inhibitors [41]. Both H5N1 and H5N5 viruses carried a proline at position 28, which, according to various studies, either did not affect baloxavir susceptibility [42] or slightly increased the baloxavir EC_50_ value in H3N2 viruses [43] without meeting the WHO criteria for reduced susceptibility [41]. One of the NA-ea5 H5N1 outbreaks was caused by a virus bearing PA-A37S, and the other was associated with a PA-I38V-bearing strain. Other substitutions at these positions (A37T and I38F/M/T) were previously associated with reduced susceptibility to baloxavir [21,44]. The PA-A37S and I38V substitutions have also been detected at low frequency in H5N1 strains in the USA; however, their phenotypic relevance in the H5N1 background remains unclear [22]. Global surveillance data showed that only 0.54% of HPAI H5N1 viruses collected in 2022–2023 with publicly available PA sequences carried genetic markers of reduced susceptibility to baloxavir [40].

## 4. Discussion

In this study, we analyzed the prevalence of genetic markers associated with reduced susceptibility to PA inhibitors and M2 blockers, as well as markers of reduced inhibition by NAIs in clade 2.3.4.4b H5N1 and H5N5 influenza viruses isolated in Russia in 2021–2022. In addition, the inhibitory activity of oseltamivir, zanamivir, and peramivir against isolated strains was investigated.

Functional NIA showed that none of the tested isolates exhibited a ≥10-fold increase in oseltamivir, zanamivir, or peramivir IC_50_ values compared to the corresponding subtype medians, indicating normal levels of inhibition according to the WHO criteria [36].

Median levels of drug inhibitory activity were comparable for tested avian H5Nx viruses and a susceptible seasonal A(H1N1)pdm09 strain, except for slightly decreased inhibition of H5N1 viruses by oseltamivir (4.3-fold) and H5N5 viruses by zanamivir (3.4-fold) [36]. At the same time, both H5N1 and H5N5 viruses were inhibited at lower concentrations of oseltamivir, zanamivir, and peramivir compared to the typical B/Victoria strain. Similar inhibitory profiles were demonstrated for clade 2.3.4.4b H5N1 influenza viruses from North America [21,45]. Given the limited data on the drug’s clinical efficacy against zoonotic influenza [46], the comparable IC_50_ values observed for avian and seasonal influenza A viruses suggest that NAIs may be useful in treating zoonotic and pandemic influenza analogous to seasonal epidemics. Nevertheless, the pathogenicity of zoonotic strains depends on multiple viral genes [47] and can significantly differ from that of seasonal strains, thereby affecting treatment efficacy. However, a number of observational studies have shown that early oseltamivir administration is linked to improved patient outcomes [18], warranting its use for the prophylaxis and treatment of zoonotic influenza [48,49].

Phylogenetic analysis of NA sequences showed that the majority of analyzed H5N1 viruses belonged to various subclades within the X1 genetic group, while only one outbreak was caused by a virus whose NA segment clustered separately (the X2 group). These NA groups presumably evolved independently from Eurasian LPAI HxN1 viruses and shared only 95.52–97.23% amino acid sequence identity, harboring a subset of AASs, including those at positions previously linked to altered enzymatic activity (222) [50,51] or drug susceptibility (430) [52]. Nevertheless, median oseltamivir, zanamivir, and peramivir IC_50_ values differed by no more than 1.2-fold between the groups, suggesting that the existing differences did not result in significant changes in the NA inhibition profile.

Pairwise comparisons of various X1 subclades showed that even though the difference in their NAIs MedIC_50_ values was statistically significant in several cases, it mostly remained minor (<2-fold), suggesting limited biological relevance (Table S5). There were 2 instances with a more pronounced difference. First, the zanamivir MedIC_50_ value of the ea3.2 subclade was 2.1-fold higher than that of the ea3.1 subclade. Although modest, this difference indicated that baseline inhibitory activity of zanamivir slightly differed between these subclades, which should be considered when applying the WHO criteria [36].

Second, viruses of the group ea5.1 bearing NA-Y253H and T452I AASs demonstrated a 29.3-fold decrease in oseltamivir IC_50_ values compared to the ea5 strains, while the difference in zanamivir and peramivir values was fairly small. Previously, the presence of an NA-Y253H substitution in clade 1 H5N1 viruses was associated with a 7–30-fold decrease in oseltamivir IC_50_ values compared to clades 2 [53], 2.3.2.1, and 2.3.4 [52], as well as other H5N1 viruses lacking this substitution [54]. Reverse genetics studies and comparison of sequence-matched strains showed that an NA-Y253H substitution alone resulted in an approximately 10–22-fold decrease in oseltamivir IC_50_ values [52,55].

Previously published structural analysis of a wild-type N1 neuraminidase showed that a tyrosine at position 253 forms a hydrogen bond with His275, which in turn forms a hydrogen bond with Glu277 [56]. Other researchers suggested that the Y253H substitution results in the loss of the 253–275 hydrogen bond, facilitating the reorientation of Glu277 needed for oseltamivir binding. They substantiated this by functional studies demonstrating that H5N1 viruses with the NA-Y253H AAS exhibited an approximately tenfold increase in oseltamivir binding without changes in zanamivir affinity [57]. Hypothetically, analogous mechanisms could be involved in the increased inhibition of the ea5.1 viruses by oseltamivir identified in our study. However, further experiments are needed to understand the role of the Y253H and T452I substitutions in the background of the 2.3.4.4b clade H5N1 virus neuraminidase.

Excluding the Russian ea5.1 strains detected in 2021–2022 and described here, analysis of global H5N1 2.3.4.4b NA sequences from the GISAID database (2020–2025; *n* = 34,543) revealed that only 0.07% (25 strains) harbored an NA-Y253H AAS and 0.01% (4 strains) carried a Y253S AAS, with all others retaining the wild-type 253Y residue (Tables S7 and S8). Only one Y253H strain (A/chicken/Belgorod/283-1V/2023, detected in January 2023 in European Russia) clustered with our ea5.1 group. Phylogenetic analysis showed that all other Y253H/S strains clustered separately across the NA tree, suggesting their independent origins due to sporadic mutations (Figure S1).

Our study suggests that accumulation of point mutations in the ea5 NA segment led to the emergence of subclade ea5.1, defined by NA-Y253H and T452I substitutions. First detected in European Russia in late May 2022, viruses of this subclade caused seven outbreaks across five regions in European and Ural Russia through mid-September 2022. Following the final registered outbreak in European Russia in January 2023 [25], the ea5.1 viruses were no longer detected in the available surveillance data. These findings demonstrate that viral lineages with altered levels of NA inhibition can emerge and circulate locally. Although the subclade identified here exhibited increased inhibition by oseltamivir, similar mechanisms (sometimes combined with reassortment) could yield fitness-competent variants with reduced antiviral susceptibility [58,59]. Such strains could spread widely and pose a major epidemiological threat, highlighting the critical need for continuous antiviral susceptibility monitoring.

Screening of influenza 2.3.4.4b H5N1 viruses isolated in Russia in 2021–2022 did not find any genetic markers of reduced or highly reduced inhibition by NAIs listed by the WHO [37]. However, a subset of viruses had alternative amino acid residues at analyzed positions.

Previously, an NA-V116A substitution was associated with RI by oseltamivir and zanamivir [60]. In our study, we identified three ea3.2 and six ea5 isolates bearing an NA-V116I substitution. In the ea3.2 group, one strain bearing a V116I substitution was an upper outlier with slightly elevated oseltamivir (1.5-fold) and zanamivir (1.75-fold) IC_50_ values relative to the group medians. In the ea5 group, four strains bearing a V116I substitution were upper outliers in zanamivir analysis (an approximately 1.4-fold IC_50_ increase). Despite these minor shifts, all V116I-bearing strains retained normal inhibition by all three tested NAIs according to the WHO criteria [37]. This aligns with recent data on recombinant neuraminidases of 2.3.4.4b strains of B3.13 and D1.1 genotypes from the United States, where NA-V116I conferred only a ~2-fold increase in zanamivir IC_50_ [22].

Among the studied ea3.2 isolates, three carried an NA-I117M substitution, and two harbored an NA-Q136P AAS. Previously, NA-I117T was linked to reduced inhibition by oseltamivir and zanamivir in clade 2.2, but not in clade 2.3.4.4b H5N1 viruses [21,61]. Similarly, NA-Q136L was reported to reduce inhibition by both these drugs in H5N1 strains [62]. In our analysis, isolates bearing either NA-I117M or NA-Q136P exhibited normal NA inhibition by oseltamivir, zanamivir, and peramivir, and were not classified as outliers in the ea3.2 group.

Previously, an NA-V149A substitution in the 150-loop was suggested to be associated with a slight increase in oseltamivir (4-fold) and zanamivir (8-fold) IC_50_ values [63]. In our study, we identified one strain (A/goose/Tyumen/359-12/2021) bearing NA-V149D, which was classified as a lower outlier in oseltamivir analysis (a 1.7-fold reduction in IC_50_) and an upper outlier in peramivir analysis (a 1.8-fold increase in IC_50_). Overall, this virus exhibited only a slight divergence from the ea5 group medians.

It was previously reported that an H5N1 virus with a combination of mutations K150N, I223L, and S247N exhibited reduced inhibition by oseltamivir [60]. In another study, introduction of NA-K150N into a clade 2.2 H5N1 strain did not alter inhibition levels [55]. In our research, one virus bearing NA-K150N (A/chicken/Orenburg/46-1V/2021, ea5) was an upper outlier in zanamivir analysis, showing only a slight IC_50_ increase compared to the ea5 median (1.35-fold). The other strain had NA-K150T (A/duck/Sakhalin/80-6V/2022, ea3.2) and was not classified as an outlier for any of the tested NAIs.

A functional assay identified one ea5 strain (A/goose/Chelyabinsk/34-1V/2021) as a lower outlier in oseltamivir analysis (3.4-fold IC_50_ reduction compared to the ea5 median) and an upper outlier in peramivir analysis (1.4-fold IC_50_ increase), while its zanamivir IC_50_ remained typical for the group. This strain harbored an NA-S442I AAS. Conversely, another ea5 isolate (A/chicken/Kursk/132-1V/2021) carried NA-S442N alongside N434D and was not an outlier. An NA-S442I substitution was previously detected in two Russian 2023 H5N1 strains (A/great_cormorant/Kalmykia/330-12V/2023 and A/dalmatian_pelican/Kalmykia/330-10V/2023) that also exhibited a ~2-fold decrease in oseltamivir IC_50_ [25], though their NA belonged to a different clade and had a subset of additional AASs. Nevertheless, further studies are needed to elucidate the impact of NA-S442I on enzyme-drug interaction.

In our study, we also investigated two HPAI outbreaks caused by clade 2.3.4.4b H5N5 viruses bearing the ea6-group NA (either full-length or with a 22-codon stalk deletion). This group seemingly acquired a full-length NA segment from Eurasian LPAI HxN5 strains, later developed a 22-codon stalk deletion, and subsequently spread outside the Caspian Sea region to Europe and North America [5,64]. Functional NIA yielded comparable IC_50_ values for strains with full-length and stalk-deleted NA, which were also consistent with data obtained for American stalk-deleted ea6-group viruses [64], confirming a normal level of inhibition by NAIs for currently circulating H5N5 influenza viruses. Analysis revealed one H5N5 strain with slightly elevated zanamivir (3.9-fold) and oseltamivir (1.8-fold) IC_50_ values, which differed from other isolates by the NA-D303N and I428V substitutions (Tables S6 and S9). Previously, the NA-I427/428T substitution (N1/N5 numbering) was shown to confer RI by zanamivir and peramivir in clade 2.3.4.4b H5N1 strains of the B3.13 and D1.1 genotypes [22]. Further experiments are needed to understand the effects of the NA-D303N and I428V substitutions in the N5 context [36].

Our study has several limitations. First, the inhibitory activity of oseltamivir, zanamivir, and peramivir was assessed in an enzymatic assay; thus, the results cannot directly predict drug efficacy in animal models or clinical practice. Further animal studies combined with observational clinical research are needed to gain deeper insights into the potency of these drugs against H5N1 and H5N5 viruses. Second, genetic and phenotypic characterization was performed on strains isolated from natural sources. Therefore, relationships between genotypic and phenotypic properties were assessed observationally, and additional reverse genetics studies are needed to establish the specific effects of the discussed amino acid substitutions. Third, in this study, we did not assess the phenotypic susceptibility of H5N1 and H5N5 viruses to M2 blockers and PA inhibitors. We only performed genetic screening for known markers of reduced susceptibility. The absence of established resistance-associated substitutions does not necessarily confirm phenotypic susceptibility. Thus, further phenotypic assessment is needed to study antiviral sensitivity to these drugs.

## 5. Conclusions

In this study, using genetic and functional approaches, we performed an antiviral susceptibility surveillance of clade 2.3.4.4b H5N1 and H5N5 influenza viruses detected during influenza outbreaks in wild and domestic birds in Russia in 2021–2022. Genetic screening showed that none of the 109 H5N1 and 9 H5N5 isolated strains carried markers of reduced susceptibility to PA inhibitors and M2 blockers or reduced inhibition by NAIs. Additional assessment using a functional neuraminidase inhibition assay confirmed that all isolates exhibited normal inhibition by oseltamivir, zanamivir, and peramivir. These data are consistent with epidemiological studies from other regions of the world, showing a low prevalence of H5N1 and H5N5 viruses with markers of reduced antiviral sensitivity or signs of reduced potency of NAIs. Additional studies using cell-based assays are needed to evaluate phenotypic susceptibility of isolated strains to M2 blockers and PA inhibitors.

Our surveillance revealed a unique group of H5N1 viruses carrying an NA segment of the ea5.1 genetic group with characteristic substitutions NA-Y253H and T452I, which showed a 28.8-fold decrease in oseltamivir IC_50_ values relative to the subtype median, while zanamivir and peramivir values were comparable to other H5N1 strains. These strains transiently circulated in the European and Ural regions of Russia from May 2022 to January 2023 without further detection in publicly available surveillance data. This observation highlights that viruses exhibiting an altered level of neuraminidase inhibition by antineuraminidase drugs can sporadically emerge, underscoring the importance of ongoing antiviral susceptibility monitoring.

## Figures and Tables

**Figure 1:**
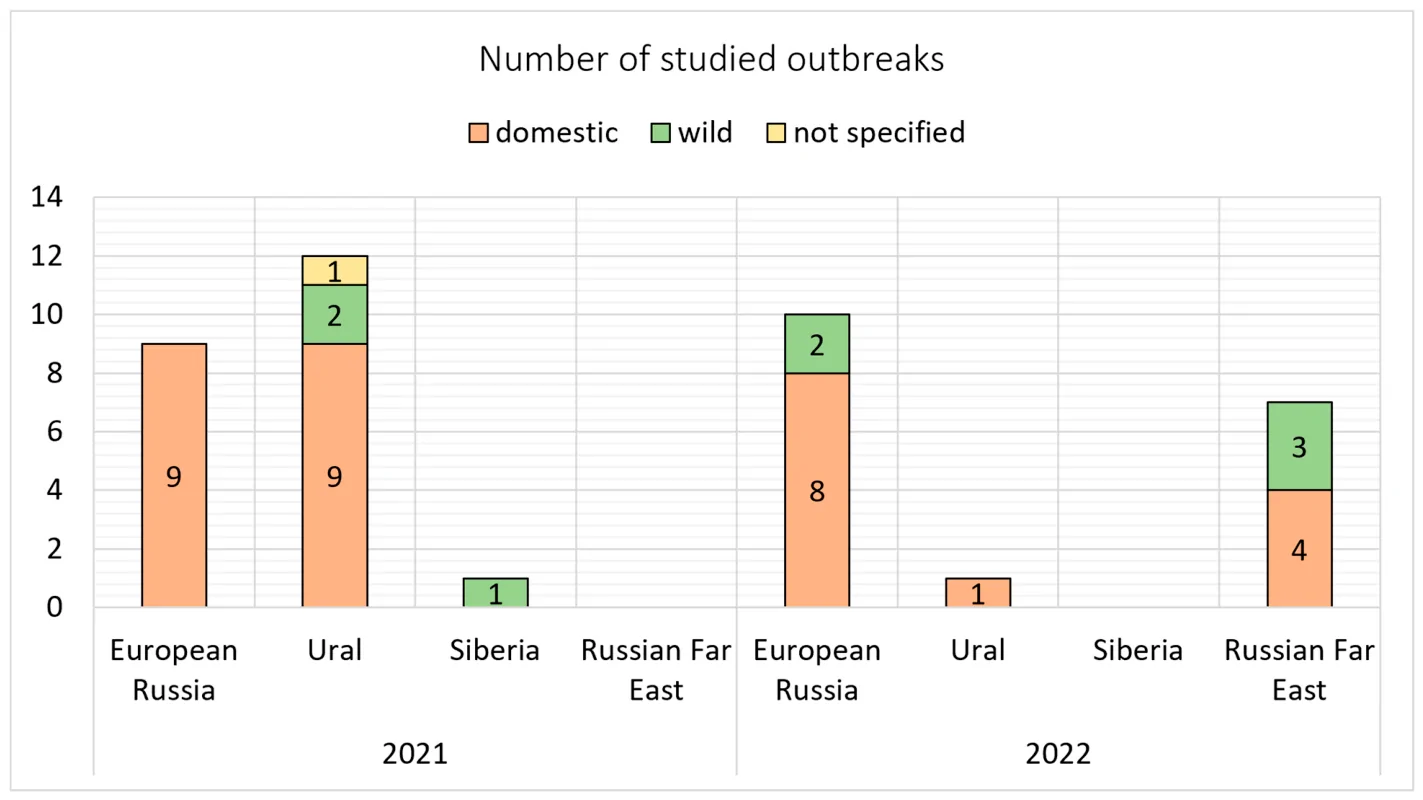
Number of clade 2.3.4.4b H5N1 influenza outbreaks in wild and domestic birds registered in Russia (2021–2022) and analyzed at the SRC VB “Vector”.

**Figure 2:**
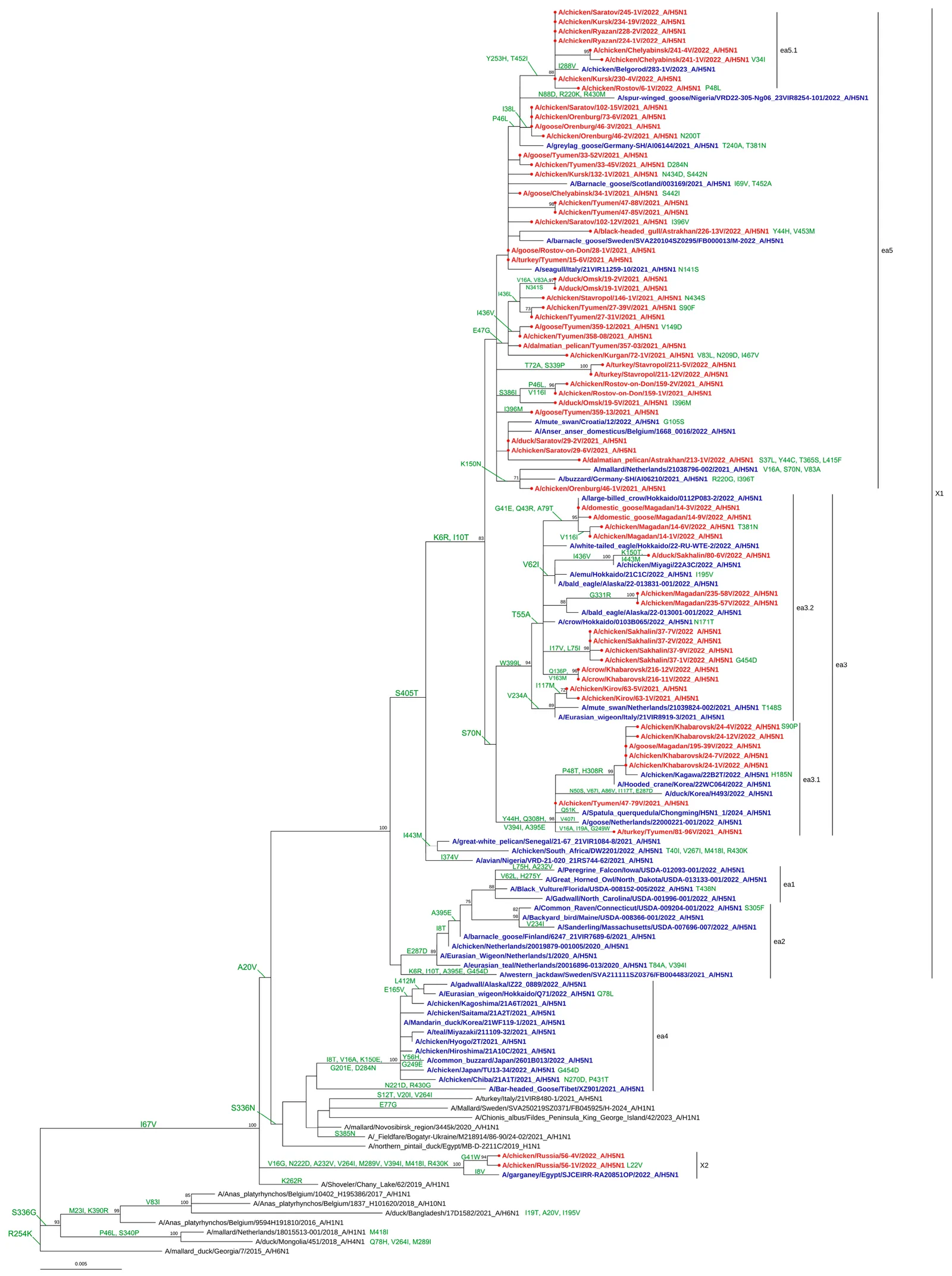
Maximum-likelihood phylogenetic tree inferred for nucleotide sequences of NA segments of A(HxN1) viruses. Influenza H5N1 viruses bearing an HA segment of the 2.3.4.4b clade are indicated in blue. Russian clade 2.3.4.4b H5N1 influenza viruses analyzed in this study are indicated in red. Amino acid substitutions relative to hypothetical ancestral strains are indicated in green on the tree branches or next to the strain names. Bootstrap values over 70 are indicated at the nodes.

**Figure 3:**
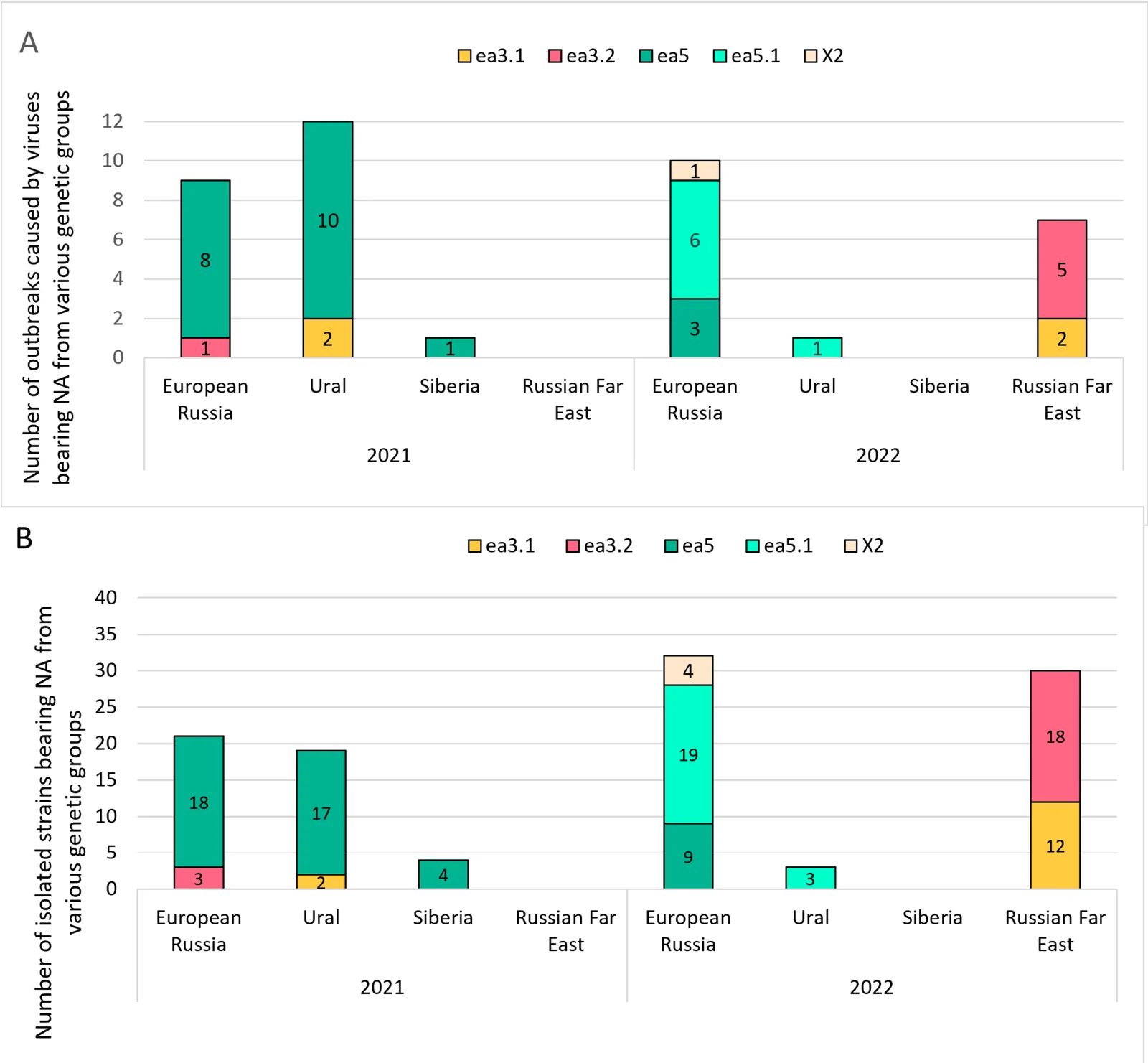
Number of clade 2.3.4.4b H5N1 influenza outbreaks (**A**) and isolated strains (**B**) belonging to different NA genetic groups analyzed across Russian regions in 2021–2022.

**Figure 4:**
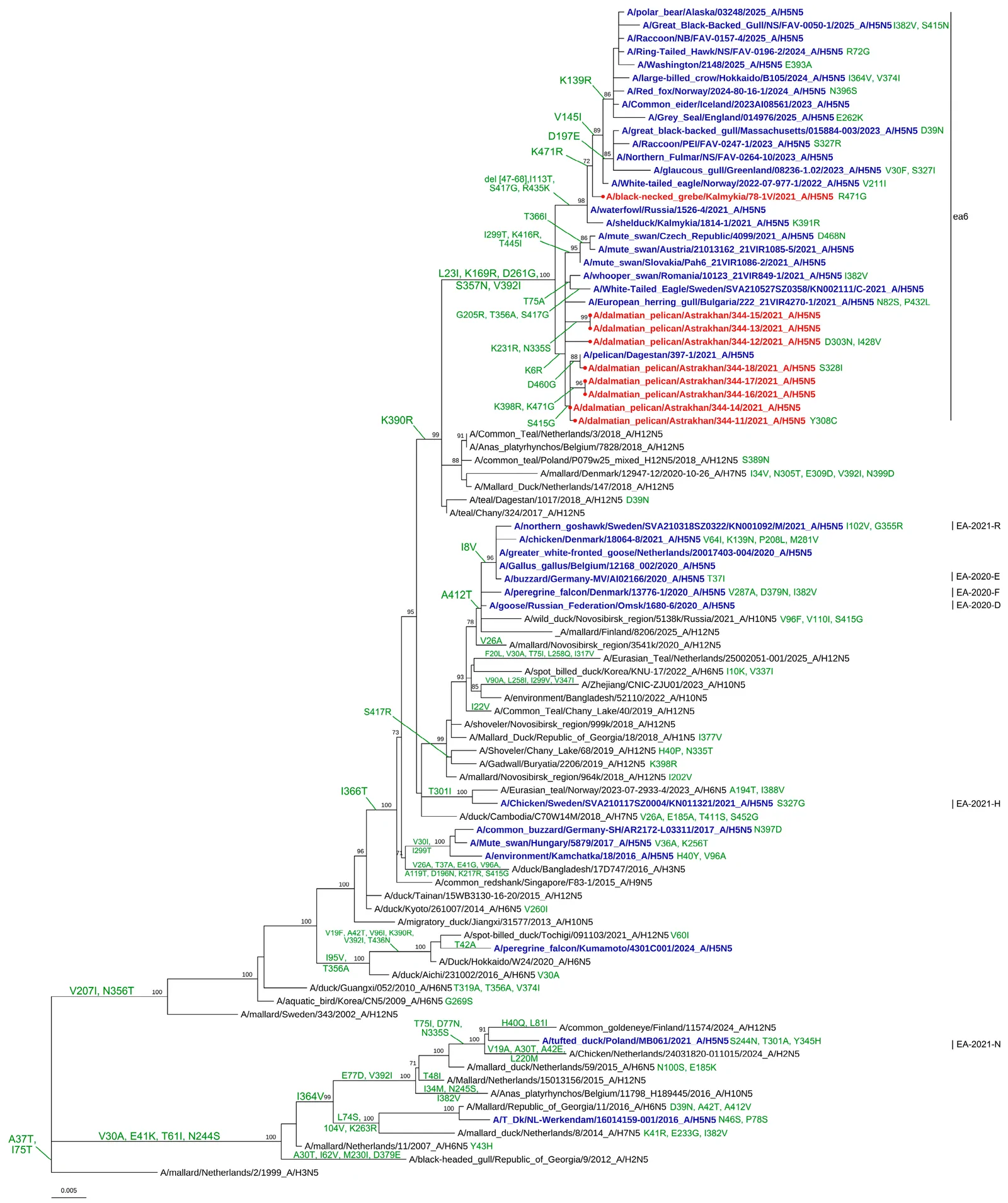
Maximum-likelihood phylogenetic tree inferred for nucleotide sequences of NA segments of A(HxN5) viruses. Clade 2.3.4.4b H5N5 influenza viruses are indicated in blue. Russian clade 2.3.4.4b H5N5 influenza viruses analyzed in this study are indicated in red. Genotype assignments according to the GENIN nomenclature (EA-XXXX-X) are given for several reference strains described in [5]. Amino acid substitutions relative to hypothetical ancestral strains are indicated in green on tree branches or next to strain names. Bootstrap values over 70 are indicated at the nodes.

**Figure 5:**
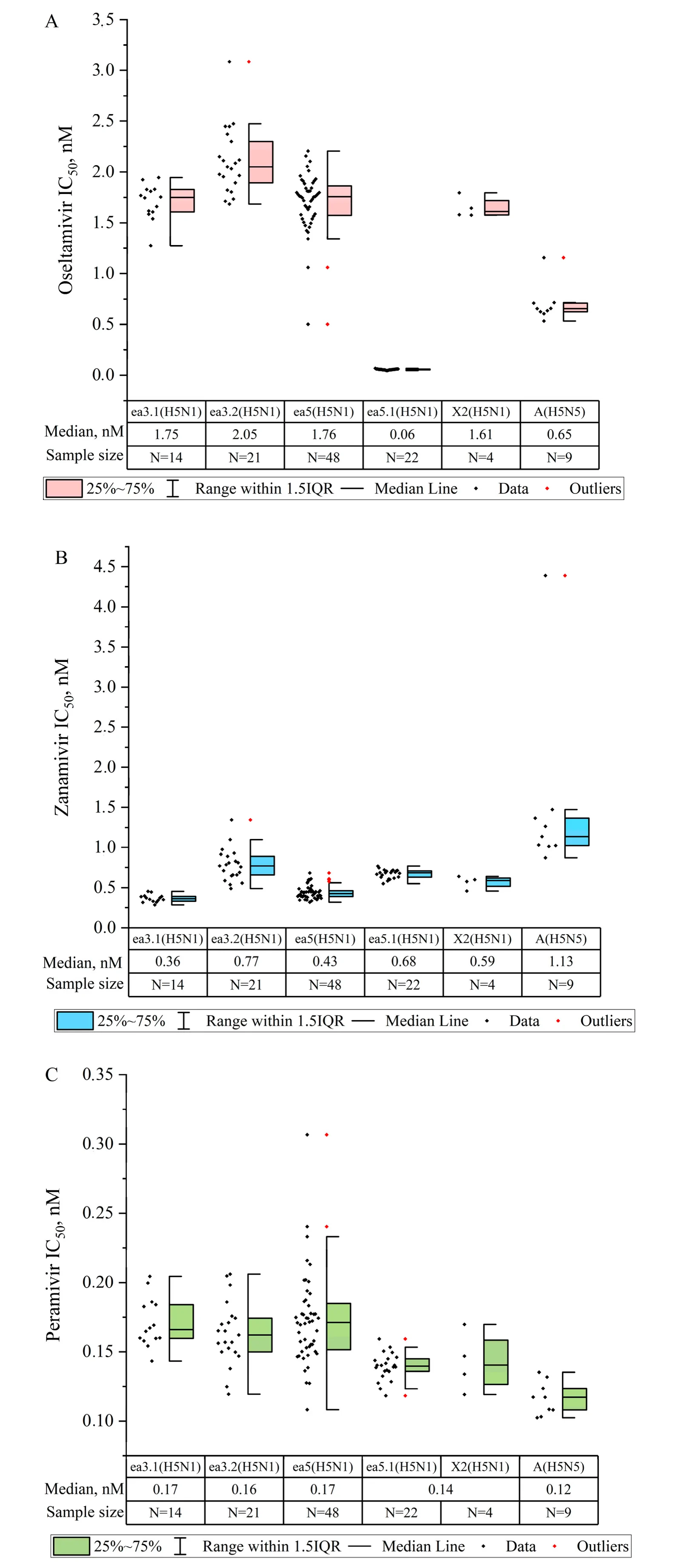
Scatter plot (left) and box-and-whisker (right) plot analysis of IC_50_ values of oseltamivir (**A**), zanamivir (**B**), and peramivir (**C**) determined for H5N5 and various NA genetic groups of H5N1 influenza viruses isolated in Russia in 2021–2022. Boxes and whiskers are plotted according to the Tukey method. The upper and lower edges of the boxes represent the 75th and 25th percentiles, respectively. Horizontal lines within the boxes represent medians of IC_50_ values determined for a particular drug and genetic group. The upper whiskers extend to the largest data point within the interval (75th percentile; 75th percentile + 1.5IQR]. The lower whiskers extend to the smallest data point within the interval [25th percentile − 1.5IQR; 25th percentile). IQR, interquartile range (the difference between the 75th and 25th percentiles). A comprehensive matrix of pairwise *p*-values from the post-hoc Dunn’s test is provided in Supplementary Table S5.

## Data Availability

The original contributions presented in this study are included in the article/Supplementary Material. Further inquiries can be directed to the corresponding author. Genetic sequences determined in this study have been deposited in the GISAID database (Table S1). The findings of this study are based on metadata associated with 173 sequences available on GISAID up to 21 August 2026, via EPI_SET_260821nb (gisaid.org), DOI: https://doi.org/10.55876/gis8.260821nb.

## Supplementary Materials

The following supporting information can be downloaded at: https://www.mdpi.com/article/10.3390/v18101126/s1, Table S1: Influenza A(H5N1) and A(H5N5) viruses analyzed in this study; Table S2: Influenza A viruses from GISAID and NCBI databases exhibiting maximal identity of NA nucleotide sequences with strain A/chicken/Russia/56-4V/2022 (according to BLAST analysis); Table S3: Analysis of A(H5N1) viruses detected in Russia in 2021–2022 for the presence of known genetic markers of reduced inhibition by neuraminidase inhibitors, amino acid substitutions at positions of structural or enzymatic importance, or substitutions at positions previously implicated in altered inhibition in other subtypes but whose effect on H5N1 remains unstudied; Table S4: Median IC_50_ values of neuraminidase inhibitors determined for A(H5N5) viruses and various genetic groups of A(H5N1) viruses compared to reference seasonal strains; Table S5: Pairwise comparison of median IC_50_ values of oseltamivir, zanamivir and peramivir determined for various subtypes and NA genetic groups; Table S6: Influenza A(H5N1) and A(H5N5) viruses identified as outliers in box-and whiskers plot analysis of oseltamivir, zanamivir and peramivir IC_50_ values; Table S7: Analysis of NA sequences of influenza A(H5N1) 2.3.4.4b viruses deposited in the GISAID database for amino acid substitutions at the 253 position (excluding ea5.1 viruses detected in 2021–2022 in Russia described in this article); Table S8: Influenza A(H5N1) 2.3.4.4b viruses bearing an NA-Y253H/S amino acid substitutions identified in the GISAID database (2020–2025, globally) (excluding ea5.1 viruses detected in 2021–2022 in Russia described in this article); Table S9: Alignment of NA amino acid sequences of representative A(H5N1) and A(H5N5) viruses detected in Russia in 2021–2022; Table S10: Influenza Virus Data Summary; Figure S1: Maximum-likelihood phylogenetic tree inferred for nucleotide sequences of NA segments of A(HxN1) viruses.

## Abbreviations

The following abbreviations are used in this manuscript:

AAS(s)	amino acid substitution(s)
AVWG	antiviral working group
BLAST	Basic Local Alignment Search Tool
BSL-3	biosafety level 3
ECE(s)	embryonated chicken egg(s)
GISAID	Global initiative on sharing all influenza data Database
HA	hemagglutinin
HPAI	highly pathogenic avian influenza
HRI	highly reduced inhibition
IC_50_	half-maximum inhibitory concentration
IQR	interquartile range
LPAI	low pathogenic avian influenza
MedIC_50_	median IC_50_
MUNANA	2′-(4-methylumbelliferyl)-α-D-N-acetylneuraminic acid
NA	neuraminidase
NAI(s)	neuraminidase inhibitor(s)
NI	normal inhibition
NCBI	National Center for Biotechnology Information
NIA	neuraminidase inhibition assay
RI	reduced inhibition
RT-PCR	reverse transcription polymerase chain reaction
SRC VB “Vector”	State Research Center of Virology and Biotechnology “Vector”, Rospotrebnadzor
WHO	World Health Organization

## References

[1] Barbezange C., Ducatez M., Fusaro A., Gonzalez J.L., Kuiken T., Melilli R., Mirinavičiūtė G., EFSA (European Food Safety Authority), ECDC (European Centre for Disease Preven-Tion and Control), EURL (European Union Reference Laboratory for Avian Influenza) (2026). Avian Influenza Overview June–August 2026. EFSA J..

[2] Lewis N.S., Banyard A.C., Whittard E., Karibayev T., Al Kafagi T., Chvala I., Byrne A., Meruyert S., King J., Harder T. (2021). Emergence and Spread of Novel H5N8, H5N5 and H5N1 Clade 2.3.4.4 Highly Pathogenic Avian Influenza in 2020. Emerg. Microbes Infect..

[3] Youk S., Torchetti M.K., Lantz K., Lenoch J.B., Killian M.L., Leyson C., Bevins S.N., Dilione K., Ip H.S., Stallknecht D.E. (2023). H5N1 Highly Pathogenic Avian Influenza Clade 2.3.4.4b in Wild and Domestic Birds: Introductions into the United States and Reassortments, December 2021–April 2022. Virology.

[4] Cui P., Shi J., Wang C., Zhang Y., Xing X., Kong H., Yan C., Zeng X., Liu L., Tian G. (2022). Global Dissemination of H5N1 Influenza Viruses Bearing the Clade 2.3.4.4b HA Gene and Biologic Analysis of the Ones Detected in China. Emerg. Microbes Infect..

[5] Fusaro A., Zecchin B., Giussani E., Palumbo E., Agüero-García M., Bachofen C., Bálint Á., Banihashem F., Banyard A.C., Beerens N. (2024). High Pathogenic Avian Influenza A(H5) Viruses of Clade 2.3.4.4b in Europe—Why Trends of Virus Evolution Are More Difficult to Predict. Virus Evol..

[6] Peacock T.P., Moncla L., Dudas G., VanInsberghe D., Sukhova K., Lloyd-Smith J.O., Worobey M., Lowen A.C., Nelson M.I. (2025). The Global H5N1 Influenza Panzootic in Mammals. Nature.

[7] Nguyen T.Q., Hutter C.R., Markin A., Thomas M., Lantz K., Killian M.L., Janzen G.M., Vijendran S., Wagle S., Inderski B. (2025). Emergence and Interstate Spread of Highly Pathogenic Avian Influenza A(H5N1) in Dairy Cattle in the United States. Science.

[8] Li C., Lyu X., Li X., Jia Z., Chai H. (2026). Current Status of Clade 2.3.4.4b H5N1 Highly Pathogenic Avian Influenza Virus Transmission in Mammals. China CDC Wkly..

[9] Garg S., Reinhart K., Couture A., Kniss K., Davis C.T., Kirby M.K., Murray E.L., Zhu S., Kraushaar V., Wadford D.A. (2025). Highly Pathogenic Avian Influenza A(H5N1) Virus Infections in Humans. N. Engl. J. Med..

[10] Jassem A.N., Roberts A., Tyson J., Zlosnik J.E.A., Russell S.L., Caleta J.M., Eckbo E.J., Gao R., Chestley T., Grant J. (2025). Critical Illness in an Adolescent with Influenza A(H5N1) Virus Infection. N. Engl. J. Med..

[11] Rolfes M.A., Kniss K., Kirby M.K., Garg S., Reinhart K., Davis C.T., Murray E.L., Wadford D.A., Harriman K., Zhu S. (2025). Human Infections with Highly Pathogenic Avian Influenza A(H5N1) Viruses in the United States from March 2024 to May 2025. Nat. Med..

[12] World Health Organization Avian Influenza A(H5N1)-Mexico. Disease Outbreak News. https://www.who.int/emergencies/disease-outbreak-news/item/2025-DON564.

[13] FAO/WHO/WOAH (2026). Updated Joint FAO/WHO/WOAH Public Health Assessment of Recent High Pathogenicity Avian Influenza A(H5) Virus Events in Animals and People. Assessment Based on Data as of 1 March 2026. 18 May 2026. https://www.who.int/publications/m/item/updated-joint-fao-who-woah-public-health-assessment-of-recent-high-pathogenicity-avian-influenza-a(h5)-virus-events-in-animals-and-people.

[14] Harrington W.N., Kackos C.M., Webby R.J. (2021). The Evolution and Future of Influenza Pandemic Preparedness. Exp. Mol. Med..

[15] Jochmans D., Laporte M., Neyts J. (2023). Antiviral Strategies for Epidemic and Pandemic Preparedness. Cell Host Microbe.

[16] Jones J.C., Yen H.L., Adams P., Armstrong K., Govorkova E.A. (2023). Influenza Antivirals and Their Role in Pandemic Preparedness. Antivir. Res..

[17] Zambon M., Myles P., Sugaya N. (2025). Use of Influenza Antivirals in Pandemic Response. J. Infect. Dis..

[18] Uyeki T.M., Belser J.A. (2025). Antivirals for Novel Influenza A Virus Infections. J. Infect. Dis..

[19] Belser J.A., Brock N., Sun X., Kieran T.J., Pulit-Penaloza J.A., Pappas C., Zeng H., Gubareva L.V., Uyeki T.M., Maines T.R. (2026). Oseltamivir and Baloxavir Monotherapy and Combination Therapy Efficacy against Clade 2.3.4.4b A(H5N1) Influenza Virus Infection in Ferrets. Commun. Biol..

[20] Andreev K., Jones J., Kandeil A., Vogel P., Webby R., Govorkova E. (2026). Baloxavir Outperforms Oseltamivir, Favipiravir, and Amantadine in Treating Lethal Influenza A(H5N1) HA Clade 2.3.4.4b Infection in Mice. Nat. Commun..

[21] Nguyen H.T., Chesnokov A., De La Cruz J., Pascua P.N.Q., Mishin V.P., Jang Y., Jones J., Di H., Ivashchenko A.A., Killian M.L. (2023). Antiviral Susceptibility of Clade 2.3.4.4b Highly Pathogenic Avian Influenza A(H5N1) Viruses Isolated from Birds and Mammals in the United States, 2022. Antivir. Res..

[22] Pascua P.N.Q., Chesnokov A.P., Nguyen H.T., Champion C., Gao R., De La Cruz J.A., Jang Y., Hatta Y., Guo Z., Uyeki T.M. (2026). Antiviral Susceptibility of Clade 2.3.4.4b Highly Pathogenic Avian Influenza A(H5N1) Viruses from Humans in the United States, October 2024 to February 2025. Emerg. Microbes Infect..

[23] Pascua P.N.Q., Chesnokov A., Nguyen H.T., Di H., De La Cruz J., Jang Y., Ivashchenko A.A., Ivachtchenko A.V., Karlsson E.A., Sar B. (2025). Antiviral Susceptibility of Influenza A(H5N1) Clade 2.3.2.1c and 2.3.4.4b Viruses from Humans, 2023–2024. Emerg. Infect. Dis..

[24] WHO Manual for the Laboratory Diagnosis and Virological Surveillance of Influenza. https://www.who.int/publications/i/item/manual-for-the-laboratory-diagnosis-and-virological-surveillance-of-influenza.

[25] Panova A.S., Kolosova N.P., Svyatchenko S.V., Goncharova N.I., Danilenko A.V., Boldyrev N.D., Shadrinova K.N., Vasiltsova N.N., Egorova M.L., Onkhonova G.S. (2025). Genetic Diversity of A(H5N1) Avian Influenza Viruses Isolated from Birds and Seals in Russia in 2023. Sci. Rep..

[26] Shu Y., McCauley J. (2017). GISAID: Global Initiative on Sharing All Influenza Data—From Vision to Reality. Eurosurveillance.

[27] Katoh K., Standley D.M. (2013). MAFFT Multiple Sequence Alignment Software Version 7: Improvements in Performance and Usability. Mol. Biol. Evol..

[28] Tamura K., Stecher G., Peterson D., Filipski A., Kumar S. (2013). MEGA6: Molecular Evolutionary Genetics Analysis Version 6.0. Mol. Biol. Evol..

[29] Minh B.Q., Schmidt H.A., Chernomor O., Schrempf D., Woodhams M.D., Von Haeseler A., Lanfear R., Teeling E. (2020). IQ-TREE 2: New Models and Efficient Methods for Phylogenetic Inference in the Genomic Era. Mol. Biol. Evol..

[30] Kalyaanamoorthy S., Minh B.Q., Wong T.K.F., Von Haeseler A., Jermiin L.S. (2017). ModelFinder: Fast Model Selection for Accurate Phylogenetic Estimates. Nat. Methods.

[31] Xu S., Dai Z., Guo P., Fu X., Liu S., Zhou L., Tang W., Feng T., Chen M., Zhan L. (2021). GgtreeExtra: Compact Visualization of Richly Annotated Phylogenetic Data. Mol. Biol. Evol..

[32] Tamuri A.U. Treesub: Annotating Ancestral Substitution on a Tree. https://github.com/tamuri/treesub.

[33] Shen W., Sipos B., Zhao L. (2024). SeqKit2: A Swiss Army Knife for Sequence and Alignment Processing. iMeta.

[34] Sayers E.W., Beck J., Bolton E.E., Brister J.R., Chan J., Connor R., Feldgarden M., Fine A.M., Funk K., Hoffman J. (2025). Database Resources of the National Center for Biotechnology Information in 2025. Nucleic Acids Res..

[35] Leang S.-K., Hurt A.C. (2017). Fluorescence-Based Neuraminidase Inhibition Assay to Assess the Susceptibility of Influenza Viruses to The Neuraminidase Inhibitor Class of Antivirals. J. Vis. Exp..

[36] WHO (2012). Meetings of the WHO Working Group on Surveillance of Influenza Antiviral Susceptibility—Geneva, November 2011 and June 2012. Wkly. Epidemiol. Rec..

[37] (2026). Expert Working Group on Antiviral Susceptibility for the WHO Global Influenza Surveillance and Response System (GISRS). Summary of Group 1 (N1, N4, N5, N8) and Group 2 (N2, N3, N6, N7, N9) Neuraminidase (NA) Amino Acid Substitutions Among Avian Influenza A Viruses Assessed for Their Effects on Inhibition by Neuraminidase Inhibitors (NAIs). Last Updated on 17 July 2026. https://cdn.who.int/media/docs/default-source/influenza/avwg/avian-nai-marker-who-table.pdf?sfvrsn=bc0d1e9a_12.

[38] GgFlu: Global Genotyping for H5 Highly Pathogenic Avian Influenza Virus. https://www.ggflu.org/.

[39] GenoFLU Classification for H5N1 HPAI 2.3.4.4b Viruses in North American Flyways. https://github.com/USDA-VS/GenoFLU.

[40] Andreev K., Jones J.C., Seiler P., Kandeil A., Turner J.C.M., Barman S., Rubrum A.M., Webby R.J., Govorkova E.A. (2024). Antiviral Susceptibility of Highly Pathogenic Avian Influenza A(H5N1) Viruses Circulating Globally in 2022–2023. J. Infect. Dis..

[41] Expert Working Group on Antiviral Susceptibility for the WHO Global Influenza Surveillance and Response System (GISRS) (2026). Baloxavir Susceptibility and Amino Acid Substitutions in Influenza Viruses. Summary of Polymerase Acidic Protein (PA) Amino Acid Substitutions Assessed for Their Effects on PA Inhibitor (PAI) Baloxavir Susceptibility. Last Updated on 30 June 2026. https://cdn.who.int/media/docs/default-source/influenza/laboratory---network/quality-assurance/antiviral-susceptibility-influenza/pa-marker-who-table_28-11-2025_updated.pdf?sfvrsn=5307d6fe_10.

[42] Govorkova E.A., Takashita E., Daniels R.S., Fujisaki S., Presser L.D., Patel M.C., Huang W., Lackenby A., Nguyen H.T., Pereyaslov D. (2022). Global Update on the Susceptibilities of Human Influenza Viruses to Neuraminidase Inhibitors and the Cap-Dependent Endonuclease Inhibitor Baloxavir, 2018–2020. Antivir. Res..

[43] Omoto S., Speranzini V., Hashimoto T., Noshi T., Yamaguchi H., Kawai M., Kawaguchi K., Uehara T., Shishido T., Naito A. (2018). Characterization of Influenza Virus Variants Induced by Treatment with the Endonuclease Inhibitor Baloxavir Marboxil. Sci. Rep..

[44] Taniguchi K., Noshi T., Omoto S., Sato A., Shishido T., Matsuno K., Okamatsu M., Krauss S., Webby R.J., Sakoda Y. (2024). The Impact of PA/I38 Substitutions and PA Polymorphisms on the Susceptibility of Zoonotic Influenza A Viruses to Baloxavir. Arch. Virol..

[45] Jones J.C., Andreev K., Fabrizio T.P., Bowman A.S., Govorkova E.A., Webby R.J. (2025). Baloxavir Improves Disease Outcomes in Mice after Intranasal or Ocular Infection with Influenza A Virus H5N1-Contaminated Cow’s Milk. Nat. Microbiol..

[46] Seida J., Barati E., Chojecki D. (2025). Antiviral Drugs for the Prevention or Treatment of Zoonotic Influenza. Can. J. Health Technol..

[47] Kuiken T., Riteau B., Fouchier R.A.M., Rimmelzwaan G.F. (2012). Pathogenesis of Influenza Virus Infections: The Good, the Bad and the Ugly. Curr. Opin. Virol..

[48] CDC (2025). Interim Guidance on the Use of Antiviral Medications for Treatment of Human Infections with Novel Influenza A Viruses Associated with Severe Human Disease. https://www.cdc.gov/bird-flu/hcp/clinicians-evaluating-patients/interim-guidance-treatment-humans.html#print.

[49] World Health Organization (2024). Clinical Practice Guidelines for Influenza.

[50] Rameix-Welti M.A., Munier S., Le Gal S., Cuvelier F., Agou F., Enouf V., Naffakh N., Van Der Werf S. (2011). Neuraminidase of 2007–2008 Influenza A(H1N1) Viruses Shows Increased Affinity for Sialic Acids Due to the D344N Substitution. Antivir. Ther..

[51] Nguyen H.T., Fry A.M., Gubareva L.V. (2012). Neuraminidase Inhibitor Resistance in Influenza Viruses and Laboratory Testing Methods. Antivir. Ther..

[52] Nguyen H.T., Nguyen T., Mishin V.P., Sleeman K., Balish A., Jones J., Creanga A., Marjuki H., Uyeki T.M., Nguyen D.H. (2013). Antiviral Susceptibility of Highly Pathogenic Avian Influenza A(H5N1) Viruses Isolated from Poultry, Vietnam, 2009–2011. Emerg. Infect. Dis..

[53] McKimm-Breschkin J.L., Selleck P.W., Usman T.B., Johnson M.A. (2007). Reduced Sensitivity of Influenza A (H5N1) to Oseltamivir. Emerg. Infect. Dis..

[54] Rameix-Welti M.A., Agou F., Buchy P., Mardy S., Aubin J.T., Véron M., Van Der Werf S., Naffakh N. (2006). Natural Variation Can Significantly Alter the Sensitivity of Influenza A (H5N1) Viruses to Oseltamivir. Antimicrob. Agents Chemother..

[55] Ilyushina N.A., Seiler J.P., Rehg J.E., Webster R.G., Govorkova E.A. (2010). Effect of Neuraminidase Inhibitor-Resistant Mutations on Pathogenicity of Clade 2.2 A/Turkey/15/06 (H5N1) Influenza Virus in Ferrets. PLoS Pathog..

[56] Russell R.J., Haire L.F., Stevens D.J., Collins P.J., Lin Y.P., Blackburn G.M., Hay A.J., Gamblin S.J., Skehel J.J. (2006). The Structure of H5N1 Avian Influenza Neuraminidase Suggests New Opportunities for Drug Design. Nature.

[57] Collins P.J., Haire L.F., Lin Y.P., Liu J., Russell R.J., Walker P.A., Skehel J.J., Martin S.R., Hay A.J., Gamblin S.J. (2008). Crystal Structures of Oseltamivir-Resistant Influenza Virus Neuraminidase Mutants. Nature.

[58] Meijer A., Lackenby A., Hungnes O., Lina B., Van Der Werf S., Schweiger B., Opp M., Paget J., Van De Kassteele J., Hay A. (2009). Oseltamivir-Resistant Influenza Virus A (H1N1), Europe, 2007–08 Season. Emerg. Infect. Dis..

[59] Zaraket H., Saito R., Suzuki Y., Suzuki Y., Caperig-Dapat I., Dapat C., Shabana I.I., Baranovich T., Suzuki H. (2010). Genomic Events Contributing to the High Prevalence of Amantadine-Resistant Influenza A/H3N2. Antivir. Ther..

[60] Boltz D.A., Douangngeun B., Phommachanh P., Sinthasak S., Mondry R., Obert C., Seiler P., Keating R., Suzuki Y., Hiramatsu H. (2010). Emergence of H5N1 Avian Influenza Viruses with Reduced Sensitivity to Neuraminidase Inhibitors and Novel Reassortants in Lao People’s Democratic Republic. J. Gen. Virol..

[61] Kode S.S., Pawar S.D., Tare D.S., Keng S.S., Hurt A.C., Mullick J. (2019). A Novel I117T Substitution in Neuraminidase of Highly Pathogenic Avian Influenza H5N1 Virus Conferring Reduced Susceptibility to Oseltamivir and Zanamivir. Vet. Microbiol..

[62] Hurt A.C., Lowther S., Middleton D., Barr I.G. (2010). Assessing the Development of Oseltamivir and Zanamivir Resistance in A(H5N1) Influenza Viruses Using a Ferret Model. Antivir. Res..

[63] Naughtin M., Dyason J.C., Mardy S., Sorn S., Von Itzstein M., Buchy P. (2011). Neuraminidase Inhibitor Sensitivity and Receptor-Binding Specificity of Cambodian Clade 1 Highly Pathogenic H5N1 Influenza Virus. Antimicrob. Agents Chemother..

[64] Erdelyan C.N.G., Kandeil A., Signore A.V., Jones M.E.B., Vogel P., Andreev K., Bøe C.A., Gjerset B., Alkie T.N., Yason C. (2024). Multiple Transatlantic Incursions of Highly Pathogenic Avian Influenza Clade 2.3.4.4b A(H5N5) Virus into North America and Spillover to Mammals. Cell Rep..

