# Supplementary material for: Antiviral Susceptibility of Clade 2.3.4.4b H5N1 and H5N5 Influenza Viruses Detected in Birds in Russia During 2021–2022
Source: Viruses. 2026 Oct 10;18(10):1126. doi: 10.3390/v18101126 (PMC13709846; doi:10.3390/v18101126)
Supplement: Supplementary file 1 [file viruses-18-01126-s001.zip › Figure S1.PDF]

Antiviral susceptibility of clade 2.3.4.4b H5N1 and H5N5 influenza viruses detected in birds in Russia during 2021-2022

Svetlana V. Svyatchenko, Natalia I. Goncharova, Ivan M. Susloparov, Natalia N. Vasiltsova, Natalia P. Kolosova, Marina L. Egorova, Tatyana V. Tregubchak, Natalia V. Danilchenko, Tatyana N. Ilyicheva, Vasiliy Y. Marchenko

Figure S1. Maximum-likelihood phylogenetic tree inferred for nucleotide sequences of NA segments of A(HxN1) viruses.

Influenza 2.3.4.4b A(H5N1) viruses bearing an NA-Y253H substitutions identified in GISAID database are indicated in pink (aside from the ones described in this article).

Influenza 2.3.4.4b A(H5N1) viruses bearing an NA-Y253H substitutions identified in Russia in 2021-2022 and described in this article are indicated in green.

Influenza 2.3.4.4b A(H5N1) viruses bearing an NA-Y253S substitutions identified in GISAID database are indicated in blue.

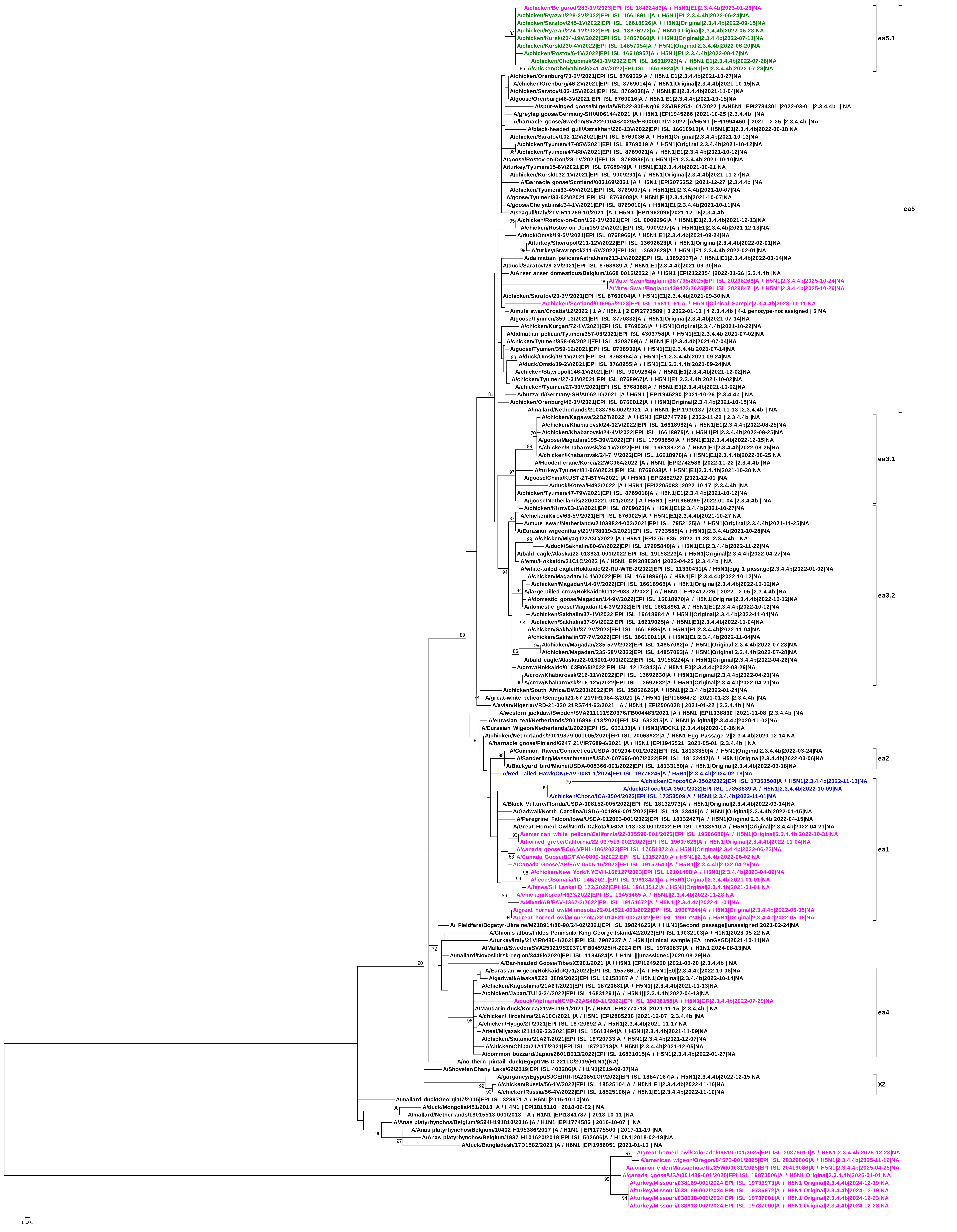
A/chicken/Belgorod/283-1V/2023|EPI_ISL_18462486|A_/_H5N1|E1|2.3.4.4b|2023-01-26|NA
A/chicken/Ryazan/228-2V/2022|EPI_ISL_16618911|A_/_H5N1|E1|2.3.4.4b|2022-06-24|NA
A/chicken/Saratov/245-1V/2022|EPI_ISL_16618926|A_/_H5N1|Original|2.3.4.4b|2022-09-15|NA
A/chicken/Ryazan/224-1V/2022|EPI_ISL_13876272|A_/_H5N1|Original|2.3.4.4b|2022-05-28|NA
A/chicken/Kursk/234-19V/2022|EPI_ISL_14857060|A_/_H5N1|Original|2.3.4.4b|2022-07-11|NA
A/chicken/Kursk/230-4V/2022|EPI_ISL_14857054|A_/_H5N1|Original|2.3.4.4b|2022-06-20|NA
A/chicken/Rostov/6-1V/2022|EPI_ISL_16618957|A_/_H5N1|E1|2.3.4.4b|2022-08-17|NA
A/chicken/Chelyabinsk/241-1V/2022|EPI_ISL_16618923|A_/_H5N1|E1|2.3.4.4b|2022-07-28|NA
A/chicken/Chelyabinsk/241-4V/2022|EPI_ISL_16618924|A_/_H5N1|E1|2.3.4.4b|2022-07-28|NA
ea5.1
A/chicken/Orenburg/73-6V/2021|EPI_ISL_8769029|A_/_H5N1|E1|2.3.4.4b|2021-10-27|NA
A/chicken/Orenburg/46-2V/2021|EPI_ISL_8769014|A_/_H5N1|Original|2.3.4.4b|2021-10-15|NA
A/chicken/Saratov/102-15V/2021|EPI_ISL_8769038|A_/_H5N1|E1|2.3.4.4b|2021-11-04|NA
A/goose/Orenburg/46-3V/2021|EPI_ISL_8769016|A_/_H5N1|E1|2.3.4.4b|2021-10-15|NA
A/spur-winged goose/Nigeria/VRD22-305-Ng06 23VIR8254-101/2022 | A/H5N1 |EPI2784301 |2022-03-01 |2.3.4.4b | NA
A/greylag goose/Germany-SH/AI06144/2021 |A / H5N1 |EPI1945266 |2021-10-25 |2.3.4.4b |NA
A/barnacle goose/Sweden/SVA220104SZ0295/FB000013/M-2022 |A/H5N1 |EPI1994460 | 2021-12-25 |2.3.4.4b |NA
A/black-headed gull/Astrakhan/226-13V/2022|EPI_ISL_16618910|A_/_H5N1|E1|2.3.4.4b|2022-06-18|NA
A/chicken/Saratov/102-12V/2021|EPI_ISL_8769036|A_/_H5N1|Original|2.3.4.4b|2021-10-13|NA
A/chicken/Tyumen/47-85V/2021|EPI_ISL_8769019|A_/_H5N1|Original|2.3.4.4b|2021-10-12|NA
A/chicken/Tyumen/47-88V/2021|EPI_ISL_8769021|A_/_H5N1|E1|2.3.4.4b|2021-10-12|NA
A/goose/Rostov-on-Don/28-1V/2021|EPI_ISL_8768986|A_/_H5N1|E1|2.3.4.4b|2021-10-10|NA
A/turkey/Tyumen/15-6V/2021|EPI_ISL_8768949|A_/_H5N1|E1|2.3.4.4b|2021-09-21|NA
A/chicken/Kursk/132-1V/2021|EPI_ISL_9009291|A_/_H5N1|Original|2.3.4.4b|2021-11-27|NA
A/Barnacle goose/Scotland/003169/2021 |A / H5N1 |EPI2076252 |2021-12-27 |2.3.4.4b |NA
A/chicken/Tyumen/33-45V/2021|EPI_ISL_8769007|A_/_H5N1|E1|2.3.4.4b|2021-10-07|NA
A/goose/Tyumen/33-52V/2021|EPI_ISL_8769008|A_/_H5N1|E1|2.3.4.4b|2021-10-07|NA
A/goose/Chelyabinsk/34-1V/2021|EPI_ISL_8769010|A_/_H5N1|E1|2.3.4.4b|2021-10-11|NA
A/seagull/Italy/21VIR11259-10/2021 |A / H5N1 |EPI1962096|2021-12-15|2.3.4.4b
A/chicken/Rostov-on-Don/159-1V/2021|EPI_ISL_9009296|A_/_H5N1|E1|2.3.4.4b|2021-12-13|NA
A/chicken/Rostov-on-Don/159-2V/2021|EPI_ISL_9009297|A_/_H5N1|E1|2.3.4.4b|2021-12-13|NA
A/duck/Omsk/19-5V/2021|EPI_ISL_8768966|A_/_H5N1|E1|2.3.4.4b|2021-09-24|NA
A/turkey/Stavropol/211-12V/2022|EPI_ISL_13692623|A_/_H5N1|Original|2.3.4.4b|2022-02-01|NA
A/turkey/Stavropol/211-5V/2022|EPI_ISL_13692628|A_/_H5N1|E1|2.3.4.4b|2022-02-01|NA
A/dalmatian_pelican/Astrakhan/213-1V/2022|EPI_ISL_13692637|A_/_H5N1|E1|2.3.4.4b|2022-03-14|NA
A/duck/Saratov/29-2V/2021|EPI_ISL_8768989|A_/_H5N1|E1|2.3.4.4b|2021-09-30|NA
A/Anser anser domesticus/Belgium/1668 0016/2022 |A / H5N1 |EPI2122854 |2022-01-26 |2.3.4.4b |NA
A/Mute_Swan/England/387795/2025|EPI_ISL_20298268|A_/_H5N1|2.3.4.4b|2025-10-24|NA
A/Mute_Swan/England/429423/2025|EPI_ISL_20298471|A_/_H5N1|2.3.4.4b|2025-10-26|NA
A/chicken/Saratov/29-6V/2021|EPI_ISL_8769004|A_/_H5N1|E1|2.3.4.4b|2021-09-30|NA
A/chicken/Scotland/006955/2023|EPI_ISL_16811191|A_/_H5N1|Clinical_Sample|2.3.4.4b|2023-01-11|NA
A/mute swan/Croatia/12/2022 | 1 A / H5N1 | 2 EPI2773589 | 3 2022-01-11 | 4 2.3.4.4b | 4-1 genotype-not assigned | 5 NA
A/goose/Tyumen/359-13/2021|EPI_ISL_3770832|A_/_H5N1|Original|2.3.4.4b|2021-07-14|NA
A/chicken/Kurgan/72-1V/2021|EPI_ISL_8769026|A_/_H5N1|Original|2.3.4.4b|2021-10-22|NA
A/dalmatian_pelican/Tyumen/357-03/2021|EPI_ISL_4303758|A_/_H5N1|E1|2.3.4.4b|2021-07-02|NA
A/chicken/Tyumen/358-08/2021|EPI_ISL_4303759|A_/_H5N1|E1|2.3.4.4b|2021-07-04|NA
A/goose/Tyumen/359-12/2021|EPI_ISL_8768939|A_/_H5N1|E1|2.3.4.4b|2021-07-14|NA
A/duck/Omsk/19-1V/2021|EPI_ISL_8768954|A_/_H5N1|E1|2.3.4.4b|2021-09-24|NA
A/duck/Omsk/19-2V/2021|EPI_ISL_8768955|A_/_H5N1|E1|2.3.4.4b|2021-09-24|NA
A/chicken/Stavropol/146-1V/2021|EPI_ISL_9009294|A_/_H5N1|E1|2.3.4.4b|2021-12-02|NA
A/chicken/Tyumen/27-31V/2021|EPI_ISL_8768967|A_/_H5N1|E1|2.3.4.4b|2021-10-02|NA
A/chicken/Tyumen/27-39V/2021|EPI_ISL_8768968|A_/_H5N1|E1|2.3.4.4b|2021-10-02|NA
A/buzzard/Germany-SH/AI06210/2021 |A / H5N1 | EPI1945290 |2021-10-26 |2.3.4.4b | NA
A/chicken/Orenburg/46-1V/2021|EPI_ISL_8769012|A_/_H5N1|Original|2.3.4.4b|2021-10-15|NA
A/mallard/Netherlands/21038796-002/2021 |A / H5N1 |EPI1930137 |2021-11-13 |2.3.4.4b | NA
ea5
A/chicken/Kagawa/22B2T/2022 |A / H5N1 |EPI2747729 | 2022-11-22 | 2.3.4.4b |NA
A/chicken/Khabarovsk/24-12V/2022|EPI_ISL_16618982|A_/_H5N1|E1|2.3.4.4b|2022-08-25|NA
A/chicken/Khabarovsk/24-4V/2022|EPI_ISL_16618975|A_/_H5N1|E1|2.3.4.4b|2022-08-25|NA
A/goose/Magadan/195-39V/2022|EPI_ISL_17995850|A_/_H5N1|E1|2.3.4.4b|2022-12-15|NA
A/chicken/Khabarovsk/24-1V/2022|EPI_ISL_16618972|A_/_H5N1|E1|2.3.4.4b|2022-08-25|NA
A/chicken/Khabarovsk/24-7_V/2022|EPI_ISL_16618978|A_/_H5N1|E1|2.3.4.4b|2022-08-25|NA
A/Hooded crane/Korea/22WC064/2022 |A / H5N1 |EPI2742586 |2022-11-22 |2.3.4.4b |NA
A/turkey/Tyumen/81-96V/2021|EPI_ISL_8769033|A_/_H5N1|E1|2.3.4.4b|2021-10-30|NA
A/goose/China/KUST-ZT-BTY4/2021 |A / H5N1 | EPI2882927 |2021-12-01 |NA
A/duck/Korea/H493/2022 |A / H5N1 |EPI2205083 |2022-10-17 |2.3.4.4b |NA
A/chicken/Tyumen/47-79V/2021|EPI_ISL_8769018|A_/_H5N1|E1|2.3.4.4b|2021-10-12|NA
A/goose/Netherlands/22000221-001/2022 | A / H5N1 | EPI1966269 |2022-01-04 |2.3.4.4b | NA
ea3.1
A/chicken/Kirov/63-1V/2021|EPI_ISL_8769023|A_/_H5N1|E1|2.3.4.4b|2021-10-27|NA
A/chicken/Kirov/63-5V/2021|EPI_ISL_8769025|A_/_H5N1|E1|2.3.4.4b|2021-10-27|NA
A/mute_swan/Netherlands/21039824-002/2021|EPI_ISL_7952125|A_/_H5N1|Original|2.3.4.4b|2021-11-25|NA
A/Eurasian_wigeon/Italy/21VIR8919-3/2021|EPI_ISL_7733585|A_/_H5N1|E1|2.3.4.4b|2021-10-28|NA
A/chicken/Miyagi/22A3C/2022 |A / H5N1 |EPI2751835 |2022-11-23 |2.3.4.4b | NA
A/duck/Sakhalin/80-6V/2022|EPI_ISL_17995849|A_/_H5N1|E1|2.3.4.4b|2022-11-22|NA
A/bald_eagle/Alaska/22-013831-001/2022|EPI_ISL_19158223|A_/_H5N1|Original|2.3.4.4b|2022-04-27|NA
A/emu/Hokkaido/21C1C/2022 |A / H5N1 |EPI2886384 |2022-04-25 |2.3.4.4b | NA
A/white-tailed eagle/Hokkaido/22-RU-WTE-2/2022|EPI_ISL_11330431|A_/_H5N1|egg_1_passage|2.3.4.4b|2022-01-02|NA
A/chicken/Magadan/14-1V/2022|EPI_ISL_16618960|A_/_H5N1|E1|2.3.4.4b|2022-10-12|NA
A/chicken/Magadan/14-6V/2022|EPI_ISL_16618965|A_/_H5N1|Original|2.3.4.4b|2022-10-12|NA
A/large-billed crow/Hokkaido/0112P083-2/2022 | A / H5N1 | EPI2412726 | 2022-12-05 |2.3.4.4b |NA
A/domestic_goose/Magadan/14-9V/2022|EPI_ISL_16618970|A_/_H5N1|Original|2.3.4.4b|2022-10-12|NA
A/domestic_goose/Magadan/14-3V/2022|EPI_ISL_16618961|A_/_H5N1|E1|2.3.4.4b|2022-10-12|NA
A/chicken/Sakhalin/37-1V/2022|EPI_ISL_16618984|A_/_H5N1|Original|2.3.4.4b|2022-11-04|NA
A/chicken/Sakhalin/37-9V/2022|EPI_ISL_16619025|A_/_H5N1|E1|2.3.4.4b|2022-11-04|NA
A/chicken/Sakhalin/37-2V/2022|EPI_ISL_16618986|A_/_H5N1|E1|2.3.4.4b|2022-11-04|NA
A/chicken/Sakhalin/37-7V/2022|EPI_ISL_16619011|A_/_H5N1|E1|2.3.4.4b|2022-11-04|NA
A/chicken/Magadan/235-57V/2022|EPI_ISL_14857062|A_/_H5N1|Original|2.3.4.4b|2022-07-28|NA
A/chicken/Magadan/235-58V/2022|EPI_ISL_14857063|A_/_H5N1|Original|2.3.4.4b|2022-07-28|NA
A/bald_eagle/Alaska/22-013001-001/2022|EPI_ISL_19158224|A_/_H5N1|Original|2.3.4.4b|2022-04-26|NA
A/crow/Hokkaido/0103B065/2022|EPI_ISL_12174843|A_/_H5N1|E0|2.3.4.4b|2022-03-29|NA
A/crow/Khabarovsk/216-11V/2022|EPI_ISL_13692630|A_/_H5N1|Original|2.3.4.4b|2022-04-21|NA
A/crow/Khabarovsk/216-12V/2022|EPI_ISL_13692632|A_/_H5N1|Original|2.3.4.4b|2022-04-21|NA
ea3.2
A/chicken/South_Africa/DW2201/2022|EPI_ISL_15852626|A_/_H5N1|||2.3.4.4b|2022-01-24|NA
A/great-white pelican/Senegal/21-67 21VIR1084-8/2021 |A / H5N1 |EPI1866472 |2021-01-23 |2.3.4.4b |NA
A/avian/Nigeria/VRD-21-020 21RS744-62/2021 | A / H5N1 | EPI2506028 | 2021-01-22 | 2.3.4.4b | NA
A/western jackdaw/Sweden/SVA211111SZ0376/FB004483/2021 |A / H5N1 |EPI1938830 |2021-11-08 |2.3.4.4b |NA
A/eurasian_teal/Netherlands/20016896-013/2020|EPI_ISL_632315|A_/_H5N1|original||2.3.4.4b|2020-11-02|NA
A/Eurasian_Wigeon/Netherlands/1/2020|EPI_ISL_603133|A_/_H5N1|MDCK1||2.3.4.4b|2020-10-16|NA
A/chicken/Netherlands/20019879-001005/2020|EPI_ISL_20068922|A_/_H5N1|Egg_Passage_2||2.3.4.4b|2020-12-14|NA
A/barnacle goose/Finland/6247 21VIR7689-6/2021 |A / H5N1 |EPI1945521 |2021-05-01 |2.3.4.4b | NA
A/Common_Raven/Connecticut/USDA-009204-001/2022|EPI_ISL_18133350|A_/_H5N1|Original|2.3.4.4b|2022-03-24|NA
A/Sanderling/Massachusetts/USDA-007696-007/2022|EPI_ISL_18132447|A_/_H5N1|Original|2.3.4.4b|2022-03-06|NA
A/Backyard_bird/Maine/USDA-008366-001/2022|EPI_ISL_18133150|A_/_H5N1|Original|2.3.4.4b|2022-03-18|NA
ea2
A/Red-Tailed_Hawk/ON/FAV-0081-1/2024|EPI_ISL_19776246|A_/_H5N1||2.3.4.4b|2024-02-18|NA
A/chicken/Choco/ICA-3502/2022|EPI_ISL_17353508|A_/_H5N1|2.3.4.4b|2022-11-13|NA
A/duck/Choco/ICA-3501/2022|EPI_ISL_17353839|A_/_H5N1|2.3.4.4b|2022-10-09|NA
A/chicken/Choco/ICA-3504/2022|EPI_ISL_17353509|A_/_H5N1|2.3.4.4b|2022-11-01|NA
A/Black_Vulture/Florida/USDA-008152-005/2022|EPI_ISL_18132973|A_/_H5N1|Original|2.3.4.4b|2022-03-14|NA
A/Gadwall/North_Carolina/USDA-001996-001/2022|EPI_ISL_18133445|A_/_H5N1|Original|2.3.4.4b|2022-01-15|NA
A/Peregrine_Falcon/Iowa/USDA-012093-001/2022|EPI_ISL_18132427|A_/_H5N1|Original|2.3.4.4b|2022-04-15|NA
A/Great_Horned_Owl/North_Dakota/USDA-013133-001/2022|EPI_ISL_18133510|A_/_H5N1|Original|2.3.4.4b|2022-04-21|NA
A/american_white_pelican/California/22-035599-001/2022|EPI_ISL_19606689|A_/_H5N1|Original|2.3.4.4b|2022-10-31|NA
A/horned_grebe/California/22-037519-002/2022|EPI_ISL_19607626|A_/_H5N1|Original|2.3.4.4b|2022-11-04|NA
A/canada_goose/BC/AIVPHL-186/2022|EPI_ISL_17051372|A_/_H5N1|Original|2.3.4.4b|2022-06-22|NA
A/Canada_Goose/BC/FAV-0899-1/2022|EPI_ISL_19152710|A_/_H5N1||2.3.4.4b|2022-06-02|NA
A/Canada_Goose/AB/FAV-0505-15/2022|EPI_ISL_19157540|A_/_H5N1||2.3.4.4b|2022-04-26|NA
A/chicken/New_York/NYCVH-168127/2023|EPI_ISL_19101498|A_/_H5N1||2.3.4.4b|2023-04-09|NA
A/feces/Somalia/ID_146/2021|EPI_ISL_19613471|A_/_H5N1|Orginal|2.3.4.4b|2021-01-01|NA
A/feces/Sri_Lanka/ID_172/2022|EPI_ISL_19613512|A_/_H5N1|Orginal|2.3.4.4b|2021-01-01|NA
A/chicken/Korea/H633/2022|EPI_ISL_19453465|A_/_H5N1||2.3.4.4b|2022-11-28|NA
A/Mixed/AB/FAV-1367-3/2022|EPI_ISL_19154672|A_/_H5N1||2.3.4.4b|2022-11-01|NA
A/great_horned_owl/Minnesota/22-014521-001/2022|EPI_ISL_19607244|A_/_H5N1|Original|2.3.4.4b|2022-05-05|NA
A/great_horned_owl/Minnesota/22-014521-002/2022|EPI_ISL_19607245|A_/_H5N1|Original|2.3.4.4b|2022-05-05|NA
ea1
A/_Fieldfare/Bogatyr-Ukraine/M218914/86-90/24-02/2021|EPI_ISL_19824625|A_/_H1N1|Second_passage||unassigned|2021-02-24|NA
A/Chionis_albus/Fildes_Peninsula_King_George_Island/42/2023|EPI_ISL_19032103|A_/_H1N1|2023-05-22|NA
A/turkey/Italy/21VIR8480-1/2021|EPI_ISL_7987337|A_/_H5N1|clinical_sample|EA_nonGsGD|2021-10-11|NA
A/Mallard/Sweden/SVA250219SZ0371/FB045925/H-2024|EPI_ISL_19780837|A_/_H1N1|2024-08-13|NA
A/mallard/Novosibirsk_region/3445k/2020|EPI_ISL_1184524|A_/_H1N1|||unassigned|2020-08-29|NA
A/Bar-headed Goose/Tibet/XZ901/2021 |A / H5N1 |EPI1949200 |2021-05-20 |2.3.4.4b | NA
A/Eurasian_wigeon/Hokkaido/Q71/2022|EPI_ISL_15576617|A_/_H5N1|E0||2.3.4.4b|2022-10-08|NA
A/gadwall/Alaska/IZ22_0889/2022|EPI_ISL_19158187|A_/_H5N1|Original||2.3.4.4b|2022-10-14|NA
A/chicken/Kagoshima/21A6T/2021|EPI_ISL_18720681|A_/_H5N1|||2.3.4.4b|2021-11-13|NA
A/chicken/Japan/TU13-34/2022|EPI_ISL_16831291|A_/_H5N1|||2.3.4.4b|2022-04-13|NA
A/duck/Vietnam/NCVD-22AS469-11/2022|EPI_ISL_19866158|A_/_H5N1|OR|2.3.4.4b|2022-07-29|NA
A/Mandarin duck/Korea/21WF119-1/2021 |A / H5N1 |EPI2770718 |2021-11-15 |2.3.4.4b | NA
A/chicken/Hiroshima/21A10C/2021 |A / H5N1 |EPI2885238 |2021-12-07 |2.3.4.4b |NA
A/chicken/Hyogo/2T/2021|EPI_ISL_18720692|A_/_H5N1|2.3.4.4b|2021-11-17|NA
A/teal/Miyazaki/211109-32/2021|EPI_ISL_15613494|A_/_H5N1|2.3.4.4b|2021-11-09|NA
A/chicken/Saitama/21A2T/2021|EPI_ISL_18720733|A_/_H5N1|2.3.4.4b|2021-12-07|NA
A/chicken/Chiba/21A1T/2021|EPI_ISL_18720718|A_/_H5N1|2.3.4.4b|2021-12-05|NA
A/common_buzzard/Japan/2601B013/2022|EPI_ISL_16831015|A_/_H5N1|2.3.4.4b|2022-01-27|NA
ea4
A/northern_pintail_duck/Egypt/MB-D-2211C/2019(H1N1)(NA)
A/Shoveler/Chany_Lake/62/2019|EPI_ISL_400286|A_/_H1N1|2019-09-07|NA
A/garganey/Egypt/SJCEIRR-RA20851OP/2022|EPI_ISL_18847167|A_/_H5N1|2.3.4.4b|2022-12-15|NA
A/chicken/Russia/56-1V/2022|EPI_ISL_18525104|A_/_H5N1|E1|2.3.4.4b|2022-11-10|NA
A/chicken/Russia/56-4V/2022|EPI_ISL_18525106|A_/_H5N1|E1|2.3.4.4b|2022-11-10|NA
X2
A/mallard_duck/Georgia/7/2015|EPI_ISL_328971|A_/_H6N1|2015-10-10|NA
A/duck/Mongolia/451/2018 |A / H4N1 | EPI1818110 | 2018-09-02 | NA
A/mallard/Netherlands/18015513-001/2018 | A / H1N1 |EPI1841787 | 2018-10-11 |NA
A/Anas platyrhynchos/Belgium/9594H191810/2016 |A / H1N1 |EPI1774586 | 2016-10-07 | NA
A/Anas platyrhynchos/Belgium/10402 H195386/2017 |A / H1N1 | EPI1775500 | 2017-11-19 |NA
A/Anas_platyrhynchos/Belgium/1837_H101620/2018|EPI_ISL_502606|A_/_H10N1|2018-02-19|NA
A/duck/Bangladesh/17D1582/2021 |A / H6N1 |EPI1986051 |2021-01-10 | NA
A/great_horned_owl/Colorado/06819-001/2025|EPI_ISL_20378010|A_/_H5N1|2.3.4.4b|2025-12-23|NA
A/american_wigeon/Oregon/04573-001/2025|EPI_ISL_20329806|A_/_H5N1|2.3.4.4b|2025-11-19|NA
A/common_eider/Massachusetts/25WI00081/2025|EPI_ISL_20419086|A_/_H5N1|2.3.4.4b|2025-04-25|NA
A/canada_goose/USA/001439-001/2025|EPI_ISL_19870506|A_/_H5N1|Original|2.3.4.4b|2025-01-01|NA
A/turkey/Missouri/038169-001/2024|EPI_ISL_19736973|A_/_H5N1|Original|2.3.4.4b|2024-12-19|NA
A/turkey/Missouri/038169-002/2024|EPI_ISL_19736972|A_/_H5N1|Original|2.3.4.4b|2024-12-19|NA
A/turkey/Missouri/038618-001/2024|EPI_ISL_19737001|A_/_H5N1|Original|2.3.4.4b|2024-12-23|NA
A/turkey/Missouri/038618-002/2024|EPI_ISL_19737000|A_/_H5N1|Original|2.3.4.4b|2024-12-23|NA
0,001
