# Supplementary material for: Antiviral Susceptibility of Clade 2.3.4.4b H5N1 and H5N5 Influenza Viruses Detected in Birds in Russia During 2021–2022
Source: Viruses. 2026 Oct 10;18(10):1126. doi: 10.3390/v18101126 (PMC13709846; doi:10.3390/v18101126)
Supplement: Supplementary file 1 [file viruses-18-01126-s001.zip › Supplementary tables S1-S8.pdf]

**Supplementary materials.**

**Antiviral susceptibility of clade 2.3.4.4b H5N1 and H5N5 influenza viruses detected in birds in Russia during 2021-2022**

Svetlana V. Svyatchenko, Natalia I. Goncharova, Ivan M. Susloparov, Natalia N. Vasiltsova, Natalia P. Kolosova, Marina L. Egorova, Tatyana V. Tregubchak, Natalia V. Danilchenko, Tatyana N. Ilyicheva, Vasiliy Y. Marchenko.

**Table S1. Influenza A(H5N1) and A(H5N5) viruses analyzed in this study**

| № | Strain | Subtype | GISAID identification number | Type of a sequenced sample* | HA clade | Sample collection date | NA clade |
|---|---|---|---|---|---|---|---|
| 1 | A/chicken/Russia/56-1V/2022 | H5N1 | EPI_ISL_18525104 | E1 | 2.3.4.4b | 10.11.2022 | X2 |
| 2 | A/chicken/Russia/56-2V/2022 | H5N1 | EPI_ISL_18525105 | E1 | 2.3.4.4b | 10.11.2022 | X2 |
| 3 | A/chicken/Russia/56-4V/2022 | H5N1 | EPI_ISL_18525106 | E1 | 2.3.4.4b | 10.11.2022 | X2 |
| 4 | A/chicken/Russia/56-5V/2022 | H5N1 | EPI_ISL_18525107 | E1 | 2.3.4.4b | 10.11.2022 | X2 |
| 5 | A/chicken/Tyumen/47-79V/2021 | H5N1 | EPI_ISL_8769018 | E1 | 2.3.4.4b | 12.10.2021 | ea3.1 |
| 6 | A/turkey/Tyumen/81-96V/2021 | H5N1 | EPI_ISL_8769033 | E1 | 2.3.4.4b | 30.10.2021 | ea3.1 |
| 7 | A/chicken/Khabarovsk/24-1V/2022 | H5N1 | EPI_ISL_16618972 | E1 | 2.3.4.4b | 25.08.2022 | ea3.1 |
| 8 | A/chicken/Khabarovsk/24-2V/2022 | H5N1 | EPI_ISL_16618973 | E1 | 2.3.4.4b | 25.08.2022 | ea3.1 |
| 9 | A/chicken/Khabarovsk/24-3V/2022 | H5N1 | EPI_ISL_16618974 | E1 | 2.3.4.4b | 25.08.2022 | ea3.1 |
| 10 | A/chicken/Khabarovsk/24-4V/2022 | H5N1 | EPI_ISL_16618975 | E1 | 2.3.4.4b | 25.08.2022 | ea3.1 |
| 11 | A/chicken/Khabarovsk/24-5V/2022 | H5N1 | EPI_ISL_16618976 | E1 | 2.3.4.4b | 25.08.2022 | ea3.1 |
| 12 | A/chicken/Khabarovsk/24-7_V/2022 | H5N1 | EPI_ISL_16618978 | E1 | 2.3.4.4b | 25.08.2022 | ea3.1 |
| 13 | A/chicken/Khabarovsk/24-8V/2022 | H5N1 | EPI_ISL_16618979 | E1 | 2.3.4.4b | 25.08.2022 | ea3.1 |
| 14 | A/chicken/Khabarovsk/24-9V/2022 | H5N1 | EPI_ISL_16618980 | E1 | 2.3.4.4b | 25.08.2022 | ea3.1 |
| 15 | A/chicken/Khabarovsk/24-10V/2022 | H5N1 | EPI_ISL_16618981 | E1 | 2.3.4.4b | 25.08.2022 | ea3.1 |
| 16 | A/chicken/Khabarovsk/24-12V/2022 | H5N1 | EPI_ISL_16618982 | E1 | 2.3.4.4b | 25.08.2022 | ea3.1 |
| 17 | A/goose/Magadan/195-39V/2022 | H5N1 | EPI_ISL_17995850 | E1 | 2.3.4.4b | 15.12.2022 | ea3.1 |
| 18 | A/goose/Magadan/195-40V/2022 | H5N1 | EPI_ISL_17995851 | E1 | 2.3.4.4b | 15.12.2022 | ea3.1 |
| 19 | A/chicken/Kirov/63-1V/2021 | H5N1 | EPI_ISL_8769023 | E1 | 2.3.4.4b | 27.10.2021 | ea3.2 |
| 20 | A/chicken/Kirov/63-2V/2021 | H5N1 | EPI_ISL_8769024 | E1 | 2.3.4.4b | 27.10.2021 | ea3.2 |
| 21 | A/chicken/Kirov/63-5V/2021 | H5N1 | EPI_ISL_8769025 | E1 | 2.3.4.4b | 27.10.2021 | ea3.2 |
| 22 | A/chicken/Magadan/14-1V/2022 | H5N1 | EPI_ISL_16618960 | E1 | 2.3.4.4b | 12.10.2022 | ea3.2 |
| 23 | A/domestic_goose/Magadan/14-3V/2022 | H5N1 | EPI_ISL_16618961 | E1 | 2.3.4.4b | 12.10.2022 | ea3.2 |
| 24 | A/domestic_duck/Magadan/14-4V/2022 | H5N1 | EPI_ISL_16618962 | E1 | 2.3.4.4b | 12.10.2022 | ea3.2 |
| 25 | A/chicken/Magadan/14-6V/2022 | H5N1 | EPI_ISL_16618965 | Original | 2.3.4.4b | 12.10.2022 | ea3.2 |
| | | | EPI_ISL_16618966 | E1 | | | |

| | | | | | | | |
|---|---|---|---|---|---|---|---|
| 26 | A/chicken/Magadan/14-7V/2022 | H5N1 | EPI_ISL_16618967<br>EPI_ISL_16618968 | Original<br>E1 | 2.3.4.4b | 12.10.2022 | ea3.2 |
| 27 | A/domestic_goose/Magadan/14-9V/2022 | H5N1 | EPI_ISL_16618970<br>EPI_ISL_16618971 | Original<br>E1 | 2.3.4.4b | 12.10.2022 | ea3.2 |
| 28 | A/chicken/Sakhalin/37-1V/2022 | H5N1 | EPI_ISL_16618984<br>EPI_ISL_16618985 | Original<br>E1 | 2.3.4.4b | 04.11.2022 | ea3.2 |
| 29 | A/chicken/Sakhalin/37-2V/2022 | H5N1 | EPI_ISL_16618986 | E1 | 2.3.4.4b | 04.11.2022 | ea3.2 |
| 30 | A/chicken/Sakhalin/37-3V/2022 | H5N1 | EPI_ISL_16618987 | E1 | 2.3.4.4b | 04.11.2022 | ea3.2 |
| 31 | A/chicken/Sakhalin/37-4V/2022 | H5N1 | EPI_ISL_16618988<br>EPI_ISL_16618996 | Original<br>E1 | 2.3.4.4b | 04.11.2022 | ea3.2 |
| 32 | A/chicken/Sakhalin/37-7V/2022 | H5N1 | EPI_ISL_16619011 | E1 | 2.3.4.4b | 04.11.2022 | ea3.2 |
| 33 | A/chicken/Sakhalin/37-9V/2022 | H5N1 | EPI_ISL_16619025 | E1 | 2.3.4.4b | 04.11.2022 | ea3.2 |
| 34 | A/chicken/Sakhalin/37-20V/2022 | H5N1 | EPI_ISL_16619027<br>EPI_ISL_16619028 | Original<br>E1 | 2.3.4.4b | 04.11.2022 | ea3.2 |
| 35 | A/duck/Sakhalin/80-6V/2022 | H5N1 | EPI_ISL_17995849 | E1 | 2.3.4.4b | 22.11.2022 | ea3.2 |
| 36 | A/crow/Khabarovsk/216-11V/2022 | H5N1 | EPI_ISL_13692630<br>EPI_ISL_13692631 | Original<br>E1 | 2.3.4.4b | 21.04.2022 | ea3.2 |
| 37 | A/crow/Khabarovsk/216-12V/2022 | H5N1 | EPI_ISL_13692632<br>EPI_ISL_13692633 | Original<br>E1 | 2.3.4.4b | 21.04.2022 | ea3.2 |
| 38 | A/chicken/Magadan/235-57V/2022 | H5N1 | EPI_ISL_14857062<br>EPI_ISL_16618920 | Original<br>E1 | 2.3.4.4b | 28.07.2022 | ea3.2 |
| 39 | A/chicken/Magadan/235-58V/2022 | H5N1 | EPI_ISL_14857063<br>EPI_ISL_16618921 | Original<br>E1 | 2.3.4.4b | 28.07.2022 | ea3.2 |
| 40 | A/dalmatian_pelican/Tyumen/357-03/2021 | H5N1 | EPI_ISL_4303758 | E1 | 2.3.4.4b | 02.07.2021 | ea5 |
| 41 | A/chicken/Tyumen/358-08/2021 | H5N1 | EPI_ISL_4303759 | E1 | 2.3.4.4b | 04.07.2021 | ea5 |
| 42 | A/goose/Tyumen/359-12/2021 | H5N1 | EPI_ISL_8768939 | E1 | 2.3.4.4b | 14.07.2021 | ea5 |
| 43 | A/goose/Tyumen/359-13/2021 | H5N1 | EPI_ISL_3770832<br>EPI_ISL_8768940 | Original<br>E1 | 2.3.4.4b | 14.07.2021 | ea5 |
| 44 | A/dalmatian_pelican/Tyumen/359-14/2021 | H5N1 | EPI_ISL_8768941 | E1 | 2.3.4.4b | 14.07.2021 | ea5 |
| 45 | A/turkey/Tyumen/15-6V/2021 | H5N1 | EPI_ISL_8768949 | E1 | 2.3.4.4b | 21.09.2021 | ea5 |
| 46 | A/turkey/Tyumen/15-14V/2021 | H5N1 | EPI_ISL_8768953 | E1 | 2.3.4.4b | 21.09.2021 | ea5 |
| 47 | A/duck/Omsk/19-1V/2021 | H5N1 | EPI_ISL_8768954 | E1 | 2.3.4.4b | 24.09.2021 | ea5 |
| 48 | A/duck/Omsk/19-2V/2021 | H5N1 | EPI_ISL_8768955 | E1 | 2.3.4.4b | 24.09.2021 | ea5 |
| 49 | A/duck/Omsk/19-3V/2021 | H5N1 | EPI_ISL_8768965 | E1 | 2.3.4.4b | 24.09.2021 | ea5 |
| 50 | A/duck/Omsk/19-5V/2021 | H5N1 | EPI_ISL_8768966 | E1 | 2.3.4.4b | 24.09.2021 | ea5 |
| 51 | A/chicken/Tyumen/27-31V/2021 | H5N1 | EPI_ISL_5463793<br>EPI_ISL_8768967 | Original<br>E1 | 2.3.4.4b | 02.10.2021 | ea5 |
| 52 | A/chicken/Tyumen/27-39V/2021 | H5N1 | EPI_ISL_5463794 | Original | 2.3.4.4b | 02.10.2021 | ea5 |

| | | | | | | | |
|---|---|---|---|---|---|---|---|
| | | | EPI_ISL_8768968 | E1 | | | |
| 53 | A/chicken/Tyumen/27-42V/2021 | H5N1 | EPI_ISL_5463796 | Original | 2.3.4.4b | 02.10.2021 | ea5 |
| | | | EPI_ISL_8768974 | E1 | | | |
| 54 | A/goose/Rostov-on-Don/28-1V/2021 | H5N1 | EPI_ISL_8768986 | E1 | 2.3.4.4b | 10.10.2021 | ea5 |
| 55 | A/duck/Saratov/29-02V/2021 | H5N1 | EPI_ISL_5463797 | Original | 2.3.4.4b | 30.09.2021 | ea5 |
| | | | EPI_ISL_8768989 | E1 | | | |
| 56 | A/duck/Saratov/29-03V/2021 | H5N1 | EPI_ISL_5463798 | Original | 2.3.4.4b | 30.09.2021 | ea5 |
| 57 | A/chicken/Saratov/29-06V/2021 | H5N1 | EPI_ISL_5463800 | Original | 2.3.4.4b | 30.09.2021 | ea5 |
| | | | EPI_ISL_8769004 | E1 | | | |
| 58 | A/chicken/Tyumen/33-45V/2021 | H5N1 | EPI_ISL_5463804 | Original | 2.3.4.4b | 07.10.2021 | ea5 |
| | | | EPI_ISL_8769007 | E1 | | | |
| 59 | A/goose/Tyumen/33-52V/2021 | H5N1 | EPI_ISL_5463805 | Original | 2.3.4.4b | 07.10.2021 | ea5 |
| | | | EPI_ISL_8769008 | E1 | 2.3.4.4b | | |
| 60 | A/goose/Tyumen/33-53V/2021 | H5N1 | EPI_ISL_5463806 | Original | 2.3.4.4b | 07.10.2021 | ea5 |
| | | | EPI_ISL_8769009 | E1 | 2.3.4.4b | | |
| 61 | A/goose/Chelyabinsk/34-1V/2021 | H5N1 | EPI_ISL_8769010 | E1 | 2.3.4.4b | 11.10.2021 | ea5 |
| 62 | A/chicken/Orenburg/46-1V/2021 | H5N1 | EPI_ISL_8769012 | Original | 2.3.4.4b | 15.10.2021 | ea5 |
| | | | EPI_ISL_8769013 | E1 | | | |
| 63 | A/chicken/Orenburg/46-2V/2021 | H5N1 | EPI_ISL_8769014 | Original | 2.3.4.4b | 15.10.2021 | ea5 |
| | | | EPI_ISL_8769015 | E1 | | | |
| 64 | A/goose/Orenburg/46-3V/2021 | H5N1 | EPI_ISL_8769016 | E1 | 2.3.4.4b | 15.10.2021 | ea5 |
| 65 | A/chicken/Tyumen/47-85V/2021 | H5N1 | EPI_ISL_8769019 | Original | 2.3.4.4b | 12.10.2021 | ea5 |
| | | | EPI_ISL_8769020 | E1 | | | |
| 66 | A/chicken/Tyumen/47-88V/2021 | H5N1 | EPI_ISL_8769021 | E1 | 2.3.4.4b | 12.10.2021 | ea5 |
| 67 | A/chicken/Kurgan/72-1V/2021 | H5N1 | EPI_ISL_8769026 | Original | 2.3.4.4b | 22.10.2021 | ea5 |
| | | | EPI_ISL_8769027 | E1 | | | |
| 68 | A/chicken/Orenburg/73-6V/2021 | H5N1 | EPI_ISL_8769029 | E1 | 2.3.4.4b | 27.10.2021 | ea5 |
| 69 | A/chicken/Saratov/102-12V/2021 | H5N1 | EPI_ISL_8769036 | Original | 2.3.4.4b | 13.10.2021 | ea5 |
| | | | EPI_ISL_8769037 | E1 | | | |
| 70 | A/chicken/Saratov/102-15V/2021 | H5N1 | EPI_ISL_8769038 | E1 | 2.3.4.4b | 04.11.2021 | ea5 |
| 71 | A/chicken/Kursk/132-1V/2021 | H5N1 | EPI_ISL_9009291 | Original | 2.3.4.4b | 27.11.2021 | ea5 |
| | | | EPI_ISL_9009292 | E1 | | | |
| 72 | A/chicken/Stavropol/146-1V/2021 | H5N1 | EPI_ISL_9009294 | E1 | 2.3.4.4b | 02.12.2021 | ea5 |
| 73 | A/chicken/Rostov-on-Don/159-1V/2021 | H5N1 | EPI_ISL_9009296 | E1 | 2.3.4.4b | 13.12.2021 | ea5 |
| 74 | A/chicken/Rostov-on-Don/159-2V/2021 | H5N1 | EPI_ISL_9009297 | E1 | 2.3.4.4b | 13.12.2021 | ea5 |
| 75 | A/chicken/Rostov-on-Don/159-3V/2021 | H5N1 | EPI_ISL_9009302 | E1 | 2.3.4.4b | 13.12.2021 | ea5 |
| 76 | A/chicken/Rostov-on-Don/159-4V/2021 | H5N1 | EPI_ISL_9009303 | E1 | 2.3.4.4b | 13.12.2021 | ea5 |
| 77 | A/chicken/Rostov-on-Don/159-5V/2021 | H5N1 | EPI_ISL_9009304 | E1 | 2.3.4.4b | 13.12.2021 | ea5 |

| | | | | | | | |
|---|---|---|---|---|---|---|---|
| 78 | A/chicken/Rostov-on-Don/159-7V/2021 | H5N1 | EPI_ISL_9009317 | E1 | 2.3.4.4b | 13.12.2021 | ea5 |
| 79 | A/turkey/Stavropol/211-5V/2022 | H5N1 | EPI_ISL_13692628 | E1 | 2.3.4.4b | 01.02.2022 | ea5 |
| 80 | A/turkey/Stavropol/211-7V/2022 | H5N1 | EPI_ISL_13692622 | E1 | 2.3.4.4b | 01.02.2022 | ea5 |
| 81 | A/turkey/Stavropol/211-9V/2022 | H5N1 | EPI_ISL_13692629 | E1 | 2.3.4.4b | 01.02.2022 | ea5 |
| 82 | A/turkey/Stavropol/211-12V/2022 | H5N1 | EPI_ISL_13692623 | Original | 2.3.4.4b | 01.02.2022 | ea5 |
| | | | EPI_ISL_13692624 | E1 | | | |
| 83 | A/turkey/Stavropol/211-15V/2022 | H5N1 | EPI_ISL_13692625 | E1 | 2.3.4.4b | 01.02.2022 | ea5 |
| 84 | A/turkey/Stavropol/211-18V/2022 | H5N1 | EPI_ISL_13692626 | Original | 2.3.4.4b | 01.02.2022 | ea5 |
| | | | EPI_ISL_13692627 | E1 | | | |
| 85 | A/dalmatian_pelican/Astrakhan/213-1V/2022 | H5N1 | EPI_ISL_13692637 | E1 | 2.3.4.4b | 14.03.2022 | ea5 |
| 86 | A/dalmatian_pelican/Astrakhan/213-2V/2022 | H5N1 | EPI_ISL_13692638 | E1 | 2.3.4.4b | 14.03.2022 | ea5 |
| 87 | A/black-headed_gull/Astrakhan/226-13V/2022 | H5N1 | EPI_ISL_16618910 | E1 | 2.3.4.4b | 18.06.2022 | ea5 |
| 88 | A/chicken/Ryazan/224-1V/2022 | H5N1 | EPI_ISL_13876272 | Original | 2.3.4.4b | 28.05.2022 | ea5.1 |
| | | | EPI_ISL_13876273 | E1 | 2.3.4.4b | | |
| 89 | A/chicken/Ryazan/228-2V/2022 | H5N1 | EPI_ISL_16618911 | E1 | 2.3.4.4b | 24.06.2022 | ea5.1 |
| 90 | A/chicken/Ryazan/228-3V/2022 | H5N1 | EPI_ISL_16618912 | E1 | 2.3.4.4b | 24.06.2022 | ea5.1 |
| 91 | A/chicken/Kursk/230-4V/2022 | H5N1 | EPI_ISL_14857054 | Original | 2.3.4.4b | 20.06.2022 | ea5.1 |
| | | | EPI_ISL_16618914 | E1 | | | |
| 92 | A/chicken/Kursk/230-7V/2022 | H5N1 | EPI_ISL_14857056 | Original | 2.3.4.4b | 20.06.2022 | ea5.1 |
| | | | EPI_ISL_16618915 | E1 | | | |
| 93 | A/chicken/Kursk/230-8V/2022 | H5N1 | EPI_ISL_14857057 | Original | 2.3.4.4b | 20.06.2022 | ea5.1 |
| | | | EPI_ISL_16618916 | E1 | | | |
| 94 | A/chicken/Kursk/230-10V/2022 | H5N1 | EPI_ISL_14857058 | Original | 2.3.4.4b | 20.06.2022 | ea5.1 |
| | | | EPI_ISL_16618917 | E1 | | | |
| 95 | A/chicken/Kursk/234-19V/2022 | H5N1 | EPI_ISL_14857060 | Original | 2.3.4.4b | 11.07.2022 | ea5.1 |
| | | | EPI_ISL_16618918 | E1 | | | |
| 96 | A/quail/Kursk/234-20V/2022 | H5N1 | EPI_ISL_14857061 | Original | 2.3.4.4b | 11.07.2022 | ea5.1 |
| | | | EPI_ISL_16618919 | E1 | | | |
| 97 | A/chicken/Chelyabinsk/241-1V/2022 | H5N1 | EPI_ISL_16618923 | E1 | 2.3.4.4b | 28.07.2022 | ea5.1 |
| 98 | A/chicken/Chelyabinsk/241-4V/2022 | H5N1 | EPI_ISL_16618924 | E1 | 2.3.4.4b | 28.07.2022 | ea5.1 |
| 99 | A/chicken/Chelyabinsk/241-5V/2022 | H5N1 | EPI_ISL_16618925 | E1 | 2.3.4.4b | 28.07.2022 | ea5.1 |
| 100 | A/chicken/Saratov/245-1V/2022 | H5N1 | EPI_ISL_16618926 | Original | 2.3.4.4b | 15.09.2022 | ea5.1 |
| | | | EPI_ISL_16618927 | E1 | | | |
| 101 | A/chicken/Saratov/245-2V/2022 | H5N1 | EPI_ISL_16618928 | Original | 2.3.4.4b | 15.09.2022 | ea5.1 |
| | | | EPI_ISL_16618929 | E1 | | | |
| 102 | A/chicken/Saratov/245-5V/2022 | H5N1 | EPI_ISL_16618931 | Original | 2.3.4.4b | 15.09.2022 | ea5.1 |
| | | | EPI_ISL_16618932 | E1 | | | |
| 103 | A/chicken/Saratov/245-7V/2022 | H5N1 | EPI_ISL_16618935 | Original | 2.3.4.4b | 15.09.2022 | ea5.1 |

| | | | | | | | |
|---|---|---|---|---|---|---|---|
| | | | EPI_ISL_16618950 | E1 | | | |
| 104 | A/chicken/Saratov/245-9V/2022 | H5N1 | EPI_ISL_16618951 | Original | 2.3.4.4b | 15.09.2022 | ea5.1 |
| | | | EPI_ISL_16618953 | E1 | | | |
| 105 | A/chicken/Saratov/245-11V/2022 | H5N1 | EPI_ISL_16618954 | E1 | 2.3.4.4b | 15.09.2022 | ea5.1 |
| 106 | A/chicken/Saratov/245-13V/2022 | H5N1 | EPI_ISL_16618955 | E1 | 2.3.4.4b | 15.09.2022 | ea5.1 |
| 107 | A/chicken/Rostov/6-1V/2022 | H5N1 | EPI_ISL_16618957 | E1 | 2.3.4.4b | 17.08.2022 | ea5.1 |
| 108 | A/chicken/Rostov/6-2V/2022 | H5N1 | EPI_ISL_16618958 | E1 | 2.3.4.4b | 17.08.2022 | ea5.1 |
| 109 | A/chicken/Rostov/6-3V/2022 | H5N1 | EPI_ISL_16618959 | E1 | 2.3.4.4b | 17.08.2022 | ea5.1 |
| 1 | A/black-necked_grebe/Kalmykia/78-1V/2021 | H5N5 | EPI_ISL_8769031 | Original | 2.3.4.4b | 03.11.2021 | ea6-del[47-68] |
| | | | EPI_ISL_8769032 | E1 | | | |
| 2 | A/dalmatian_pelican/Astrakhan/344-11/2021 | H5N5 | EPI_ISL_3770831 | E1 | 2.3.4.4b | 29.03.2021 | ea6 |
| 3 | A/dalmatian_pelican/Astrakhan/344-12/2021 | H5N5 | EPI_ISL_3770925 | E1 | 2.3.4.4b | 29.03.2021 | ea6 |
| 4 | A/dalmatian_pelican/Astrakhan/344-13/2021 | H5N5 | EPI_ISL_3770926 | E1 | 2.3.4.4b | 29.03.2021 | ea6 |
| 5 | A/dalmatian_pelican/Astrakhan/344-14/2021 | H5N5 | EPI_ISL_3770927 | E1 | 2.3.4.4b | 29.03.2021 | ea6 |
| 6 | A/dalmatian_pelican/Astrakhan/344-15/2021 | H5N5 | EPI_ISL_3770928 | E1 | 2.3.4.4b | 29.03.2021 | ea6 |
| 7 | A/dalmatian_pelican/Astrakhan/344-16/2021 | H5N5 | EPI_ISL_3770929 | E1 | 2.3.4.4b | 29.03.2021 | ea6 |
| 8 | A/dalmatian_pelican/Astrakhan/344-17/2021 | H5N5 | EPI_ISL_3770946 | E1 | 2.3.4.4b | 29.03.2021 | ea6 |
| 9 | A/dalmatian_pelican/Astrakhan/344-18/2021 | H5N5 | EPI_ISL_3770962 | E1 | 2.3.4.4b | 29.03.2021 | ea6 |

* - Ex- number of passages in embryonated chicken eggs

**Table S2. Influenza A viruses from GISAID and NCBI databases exhibiting maximal identity of NA nucleotide sequences with strain A/chicken/Russia/56-4V/2022 (according to BLAST analysis).**

| | Virus (database identification number, subtype, HA clade, collection date are indicated in case information is available in database) | Max Score | Total Score | Query Cover | E value | Per. ident | Acc. Len | Source of sequence |
|---|---|---|---|---|---|---|---|---|
| 1 | A/garganey/Egypt/SJCEIRR-RA20851OP/2022\|EPI_ISL_18847167\|A/H5N1\|2.3.4.4b\|2022-12-15\|NA | 2632 | 2632 | 100% | 0.0 | 99.52 | 1458 | GISAID, NCBI |
| 2 | A/Shoveler/Chany_Lake/62/2019\|EPI_ISL_400286\|A/H1N1\|2019-09-07\|NA | 2505 | 2505 | 99% | 0.0 | 98.25 | 1441 | GISAID |
| 3 | A/mallard/Novosibirsk_region/3445k/2020\|EPI_ISL_1184524\|A/H1N1\|2020-08-29\|NA | 2505 | 2505 | 99% | 0.0 | 98.25 | 1437 | GISAID |
| 4 | A/_Fieldfare/Bogatyr-Ukraine/M218914/86-90/24-02/2021\|EPI_ISL_19824625\|A/H1N1\|2021-02-24\|NA | 2516 | 2516 | 100% | 0.0 | 98.06 | 1458 | GISAID |
| 5 | A/Mandarin_duck/Korea/21WF119-1/2021\|EPI_ISL_18373217\|A/H5N1\|2.3.4.4b\|2021-11-15\|NA | 2444 | 2444 | 97% | 0.0 | 97.94 | 1410 | GISAID |
| 6 | A/turkey/Italy/21VIR9117-2/2021\|EPI_ISL_7987342\|A/H5N1\|\|EA_nonGsGD\|2021-11-04\|NA | 2507 | 2507 | 100% | 0.0 | 97.93 | 1459 | GISAID |
| 7 | A/northern pintail duck/Egypt/MB-D-2211C/2019\| A(H1N1)\|NA | 2505 | 2505 | 100% | 0.0 | 97.93 | 1458 | NCBI |
| 8 | A/turkey/Italy/21VIR8480-1/2021\|EPI_ISL_7987337\|A/H5N1\|EA_nonGsGD\|2021-10-11\|NA | 2497 | 2497 | 100% | 0.0 | 97.86 | 1457 | GISAID |
| 9 | A/Eurasian_Wigeon/Netherlands/1/2020\|EPI_ISL_603133\|A/H5N1\|2.3.4.4b\|2020-10-16\|NA | 2471 | 2471 | 99% | 0.0 | 97.83 | 1433 | GISAID |
| 10 | A/greylag_goose/Netherlands/20016582-004/2020\|EPI_ISL_632314\|A/H5N1\|2.3.4.4b\|2020-10-28\|NA | 2471 | 2471 | 99% | 0.0 | 97.83 | 1433 | GISAID |
| 11 | A/eurasian_teal/Netherlands/20016896-013/2020\|EPI_ISL_632315\|A/H5N1\|2.3.4.4b\|2020-11-02\|NA | 2471 | 2471 | 99% | 0.0 | 97.83 | 1433 | GISAID |
| 12 | A/swan/Netherlands/20017605-002/2020\|EPI_ISL_1139101\|A/H5N1\|2.3.4.4b\|2020-11-09\|NA | 2471 | 2471 | 99% | 0.0 | 97.83 | 1433 | GISAID |
| 13 | A/chicken/Hyogo/2T/2021\|EPI_ISL_18720692\|A/H5N1\|2.3.4.4b\|2021-11-17\|NA | 2471 | 2471 | 99% | 0.0 | 97.83 | 1437 | GISAID |
| 14 | A/chicken/Hiroshima/21A10C/2021\|EPI_ISL_18720751\|A/H5N1\|2.3.4.4b\|2021-12-07\|NA | 2471 | 2471 | 99% | 0.0 | 97.83 | 1437 | GISAID |
| 15 | A/chicken/Hyogo/TU4-45,46/2021\|EPI_ISL_15063423\|A/H5N1\|2.3.4.4b\|2021-11-17\|NA | 2470 | 2470 | 99% | 0.0 | 97.83 | 1430 | GISAID |
| 16 | A/teal/Miyazaki/211109-32/2021\|EPI_ISL_15613494\|A/H5N1\|2.3.4.4b\|2021-11-09\|NA | 2462 | 2462 | 99% | 0.0 | 97.83 | 1428 | GISAID |
| 17 | A/Chionis_albus/Fildes_Peninsula,_King_George_Island/42/2023\|EPI_ISL_19032103\|A/H1N1\|2023-05-22\|NA | 2495 | 2495 | 100% | 0.0 | 97.79 | 1459 | GISAID |
| 18 | A/Mallard/Sweden/SVA250219SZ0371/FB045911/V1-Mixed_infection/H-2024\|EPI_ISL_19778152\|A/H1N1\|2024-08-04\|NA | 2494 | 2494 | 100% | 0.0 | 97.79 | 1458 | GISAID |
| 19 | A/common_buzzard/Japan/2601B013/2022\|EPI_ISL_16831015\|A/H5N1\|2.3.4.4b\|2022-01-27\|NA | 2484 | 2484 | 100% | 0.0 | 97.78 | 1444 | GISAID, NCBI |
| 20 | A/chicken/Japan/TU13-34/2022\|EPI_ISL_16831291\|A/H5N1\|2.3.4.4b\|2022-04-13\|NA | 2481 | 2481 | 100% | 0.0 | 97.78 | 1442 | GISAID, NCBI |
| 21 | A/chicken/Chiba/21A1T/2021\|EPI_ISL_18720718\|A/H5N1\|2.3.4.4b\|2021-12-05\|NA | 2466 | 2466 | 99% | 0.0 | 97.77 | 1437 | GISAID |
| 22 | A/chicken/Netherlands/20019879-001005/2020\|EPI_ISL_711055\|A/H5N1\|2.3.4.4b\|2020-12-14\|NA | 2466 | 2466 | 99% | 0.0 | 97.76 | 1433 | GISAID |
| 23 | A/common_buzzard/Netherlands/21021396-002/2021\|EPI_ISL_1048240\|A/H5N1\|2.3.4.4b\|2021-01-09\|NA | 2466 | 2466 | 99% | 0.0 | 97.76 | 1433 | GISAID |

| | | | | | | | | |
|---|---|---|---|---|---|---|---|---|
| 24 | A/barnacle_goose/Netherlands/21021591-001/2021\|EPI_ISL_1048241\|A/H5N1\|2.3.4.4b\|2021-01-14\|NA | 2466 | 2466 | 99% | 0.0 | 97.76 | 1433 | GISAID |
| 25 | A/chicken/Kagoshima/21A6T/2021\|EPI_ISL_6829533\|A/H5N1\|2.3.4.4b\|2021-11-12\|NA | 2466 | 2466 | 99% | 0.0 | 97.76 | 1437 | GISAID |
| 26 | A/chicken/Hyogo/1T/2021\|EPI_ISL_18720691\|A/H5N1\|2.3.4.4b\|2021-11-17\|NA | 2466 | 2466 | 99% | 0.0 | 97.76 | 1437 | GISAID |
| 27 | A/chicken/Kagoshima/21A1T/2021\|EPI_ISL_18720695\|A/H5N1\|2.3.4.4b\|2021-11-13\|NA | 2466 | 2466 | 99% | 0.0 | 97.76 | 1437 | GISAID |
| 28 | A/chicken/Saitama/21A2T/2021\|EPI_ISL_18720733\|A/H5N1\|2.3.4.4b\|2021-12-07\|NA | 2466 | 2466 | 99% | 0.0 | 97.76 | 1436 | GISAID |
| 29 | A/chicken/Kagoshima/21C2T/2022\|EPI_ISL_18720780\|A/H5N1\|2.3.4.4b\|2022-01-13\|NA | 2466 | 2466 | 99% | 0.0 | 97.76 | 1437 | GISAID |
| 30 | A/chicken/Saitama/TU7-34,36/2021\|EPI_ISL_15063425\|A/H5N1\|2.3.4.4b\|2021-12-07\|NA | 2464 | 2464 | 99% | 0.0 | 97.76 | 1430 | GISAID |
| 31 | A/Eurasian_wigeon/Hokkaido/Q71/2022\|EPI_ISL_15576617\|A/H5N1\|2.3.4.4b\|2022-10-08\|NA | 2446 | 2446 | 98% | 0.0 | 97.75 | 1422 | GISAID |
| 32 | A/Eurasian_wigeon/Hokkaido/M184/2022\|EPI_ISL_15732766\|A/H5N1\|2.3.4.4b\|2022-10-23\|NA | 2446 | 2446 | 98% | 0.0 | 97.75 | 1422 | GISAID |
| 33 | A/gadwall/Alaska/IZ22_0889/2022\|EPI_ISL_19158187\|A/H5N1\|2.3.4.4b\|2022-10-14\|NA | 2488 | 2488 | 100% | 0.0 | 97.72 | 1454 | GISAID |
| 34 | A/northern_shoveler/Alaska/IZ22_0892/2022\|EPI_ISL_19158263\|A/H5N1\|2.3.4.4b\|2022-10-14\|NA | 2488 | 2488 | 100% | 0.0 | 97.72 | 1454 | GISAID |
| 35 | A/American_wigeon/Alaska/IZ22_0891/2022\|EPI_ISL_19158341\|A/H5N1\|2.3.4.4b\|2022-10-14\|NA | 2488 | 2488 | 100% | 0.0 | 97.72 | 1454 | GISAID |
| 36 | A/American_wigeon/Alaska/IZ22_0890/2022\|EPI_ISL_19158342\|A/H5N1\|2.3.4.4b\|2022-10-14\|NA | 2488 | 2488 | 100% | 0.0 | 97.72 | 1454 | GISAID, NCBI |

**Table S3. Analysis of A(H5N1) viruses detected in Russia in 2021–2022 for the presence of known genetic markers of reduced inhibition by neuraminidase inhibitors, amino acid substitutions at positions of structural or enzymatic importance, or substitutions at positions previously implicated in altered inhibition in other subtypes but whose effect on H5N1 remains unstudied.**

| Position in NA protein. N1 numbering corresponding to NA of: | | Amino acid residue | Number of analyzed Russian A(H5N1) isolates with an indicated amino acid residue | Isolates with alternative amino acid residues | Source of information about analyzed position | AAS previously associated with normal (NI) or altered (RI/HRI)** inhibition by various NAIs (one or more)*** |
|---|---|---|---|---|---|---|
| A/turkey/Turkey/1/2005 (H5N1 with NA stalk deletion)* | A/bald_eagle/Florida/W22-134-OP/2022 (H5N1 without NA stalk deletion)* | | | | | |
| 96 | 116 | V | 100 | | WHO table [37] | V96/116A (RI), V96/116I (NI) |
| | | I | 9 | A/chicken/Magadan/14-1V/2022, A/domestic_duck/Magadan/14-4V/2022, A/chicken/Magadan/14-6V/2022, A/chicken/Rostov-on-Don/159-1V/2021, A/chicken/Rostov-on-Don/159-2V/2021, A/chicken/Rostov-on-Don/159-3V/2021, A/chicken/Rostov-on-Don/159-4V/2021, A/chicken/Rostov-on-Don/159-5V/2021, A/chicken/Rostov-on-Don/159-7V/2021 | | |
| 97 | 117 | I | 106 | | WHO table [37] | I97/117T (NI/RI), I97/117V(NI) |
| | | M | 3 | A/chicken/Kirov/63-1V/2021, A/chicken/Kirov/63-2V/2021, A/chicken/Kirov/63-5V/2021 | | |
| 98 | 118 | R | 109 | | Active site | |
| 99 | 119 | E | 109 | | WHO table [37], Framework region | E99/119A/D/G (RI/HRI) |
| 114 | 134 | L | 109 | | [51] | |
| 116 | 136 | Q | 107 | | WHO table [37] | Q116/136L (RI/HRI) |
| | | P | 2 | A/crow/Khabarovsk/216-11V/2022, A/crow/Khabarovsk/216-12V/2022 | | |
| 127 | 147 | G | 109 | | [52] | |
| 129 | 149 | V | 108 | | WHO table [37] | V129/149A (NI) |
| | | D | 1 | A/goose/Tyumen/359-12/202 | | |
| 130 | 150 | K | 107 | | WHO table [37] | |

| | | | | | | |
|---|---|---|---|---|---|---|
| | | N | 1 | A/chicken/Orenburg/46-1V/2021 | | K130/150N+I203/223L+S227/247N (RI) |
| | | T | 1 | A/duck/Sakhalin/80-6V/2022 | | |
| 131 | 151 | D | 109 | | Active site | |
| 132 | 152 | R | 109 | | WHO table [37], Active site | R132/152G (RI/HRI) |
| 135 | 155 | Y | 109 | | [51] | |
| 136 | 156 | R | 109 | | Framework region | |
| 159 | 179 | W | 109 | | Framework region | |
| 160 | 180 | S | 109 | | Framework region | |
| 179 | 199 | D | 109 | | WHO table [37], Framework region | D179/199G (RI) |
| 190 | 210 | G | 109 | | [51] | |
| 202 | 222 | D | 4 | A/chicken/Russia/56-4V/2022, A/chicken/Russia/56-5V/2022, A/chicken/Russia/56-2V/2022, A/chicken/Russia/56-1V/2022 | [51] | |
| | | N | 105 | | | |
| 203 | 223 | I | 109 | | WHO table [37], Framework region | I203/223M (RI), I203/223V/T (NI) |
| 205 | 225 | R | 109 | | Active site | |
| 208 | 228 | E | 109 | | Framework region | |
| 214 | 234 | V | 106 | | [51] | |
| | | A | 3 | A/chicken/Kirov/63-5V/2021, A/chicken/Kirov/63-2V/2021, A/chicken/Kirov/63-1V/2021 | | |
| 224 | 244 | D | 109 | | [51] | |
| 227 | 247 | S | 109 | | WHO table [37] | S227/247N (NI/RI) |
| 233 | 253 | H | 22 | | [52] | |
| | | Y | 87 | | | |
| 255 | 275 | H | 109 | | WHO table [37] | H255/275Y (RI/HRI) |
| 257 | 277 | E | 109 | | Active site | |
| 258 | 278 | E | 109 | | Framework region | |
| 273 | 293 | R | 109 | | Active site | |

| | | | | | | |
|---|---|---|---|---|---|---|
| 274 | 294 | D | 109 | | Framework region | |
| 275 | 295 | N | 109 | | WHO table [37] | N275/295S (RI/HRI), N275/295D (NI) |
| 294 | 314 | I | 109 | | WHO table [37] | I97/117V+I294/314V (RI) |
| 310 | 330 | D | 109 | | [51] | |
| 324 | 344 | Y | 109 | | [51] | |
| 342 | 362 | T | 109 | | [51] | |
| 348 | 368 | R | 109 | | Active site | |
| 380 | 400 | S | 109 | | [51] | |
| 382 | 402 | Y | 109 | | Active site | |
| 404 | 424 | V | 109 | | [52] | |
| 405 | 425 | E | 109 | | Framework region | |
| 407 | 427 | I | 109 | | WHO table [37] | I407/427T (RI) |
| 410 | 430 | R | 105 | | [52] | |
| | | K | 4 | A/chicken/Russia/56-4V/2022, A/chicken/Russia/56-5V/2022, A/chicken/Russia/56-2V/2022, A/chicken/Russia/56-1V/2022 | | |
| 412 | 432 | K | 109 | | WHO table [37] | K412/432T (RI), K412/432R (NI) |
| 418 | 438 | T | 109 | | WHO table [37] | T418/438I/N (RI/HRI) |
| 419 | 439 | S | 109 | | WHO table [37] | S419/439G (NI) |
| * - strains used as reference for NA numbering in [37] | | | | | | |
| ** - NI – normal inhibition, RI – reduced inhibition, HRI – highly reduced inhibition | | | | | | |
| *** - according to the “Summary of Group 1 (N1, N4, N5, N8) and Group 2 (N2, N3, N6, N7, N9) Neuraminidase (NA) Amino Acid Substitutions Among Avian Influenza A Viruses Assessed for Their Effects on Inhibition by Neuraminidase Inhibitors (NAIs). Last Updated on 17 July 2026”. Expert Working Group on Antiviral Susceptibility for the WHO Global Influenza Surveillance and Response System (GISRS) [37]. | | | | | | |

**Table S4. Median $IC_{50}$ values of neuraminidase inhibitors determined for A(H5N5) viruses and various genetic groups of A(H5N1) viruses compared to reference seasonal strains.**

| **Group of viruses** | N[a] | Oseltamivir $IC_{50}$, nM | | | | | Zanamivir $IC_{50}$, nM | | | | | Peramivir $IC_{50}$, nM | | | | |
|---|---|---|---|---|---|---|---|---|---|---|---|---|---|---|---|---|
| | | Min[b] | Q1 | Median | Q3 | Max | Min | Q1 | Median | Q3 | Max | Min | Q1 | Median | Q3 | Max |
| ea3.1 NA-group (H5N1) | 14 | 1.27 | 1.60 | 1.75 | 1.83 | 1.94 | 0.28 | 0.33 | 0.36 | 0.39 | 0.45 | 0.14 | 0.16 | 0.17 | 0.18 | 0.20 |
| ea3.2 NA-group (H5N1) | 21 | 1.68 | 1.86 | 2.05 | 2.33 | 3.08 | 0.49 | 0.65 | 0.77 | 0.90 | 1.35 | 0.12 | 0.15 | 0.16 | 0.18 | 0.21 |
| ea5 NA-group (H5N1) | 48 | 0.50 | 1.57 | 1.76 | 1.87 | 2.21 | 0.32 | 0.39 | 0.43 | 0.46 | 0.68 | 0.11 | 0.15 | 0.17 | 0.19 | 0.31 |
| ea5.1 NA-group (H5N1) | 22 | 0.05 | 0.05 | 0.06 | 0.06 | 0.07 | 0.55 | 0.63 | 0.68 | 0.71 | 0.77 | 0.12 | 0.13 | 0.14 | 0.15 | 0.16 |
| X1 NA group (H5N1) | 105 | 0.05 | 1.37 | 1.75 | 1.91 | 3.08 | 0.28 | 0.39 | 0.49 | 0.68 | 1.35 | 0.11 | 0.15 | 0.16 | 0.18 | 0.31 |
| X2 NA-group (H5N1) | 4 | 1.57 | 1.57 | 1.61 | 1.76 | 1.80 | 0.46 | 0.49 | 0.59 | 0.63 | 0.64 | 0.12 | 0.12 | 0.14 | 0.16 | 0.17 |
| A(H5N1) subtype | 109 | 0.05 | 1.42 | 1.73 | 1.90 | 3.08 | 0.28 | 0.40 | 0.49 | 0.68 | 1.35 | 0.11 | 0.14 | 0.16 | 0.18 | 0.31 |
| A(H5N5) subtype | 9 | 0.53 | 0.61 | 0.65 | 0.71 | 1.15 | 0.87 | 1.02 | 1.13 | 1.42 | 4.39 | 0.10 | 0.11 | 0.12 | 0.13 | 0.14 |
| **Reference seasonal strains** | | | | Mean $IC_{50}$±SD | | | | | Mean $IC_{50}$±SD | | | | | Mean $IC_{50}$±SD | | |
| A/California/07/2009 (H1N1pdm09) | | | | 0.4 ± 0.06 | | | | | 0.34 ± 0.04 | | | | | 0.11 ± 0.02 | | |
| A/North Carolina/39/2009 (H1N1pdm09), NA-H275Y | | | | 124.96 ± 17.56 | | | | | 0.43 ± 0.06 | | | | | 41.7 ± 4.85 | | |
| B/Austria/1359417/2021 (B/Victoria) | | | | 31.57 ± 6.09 | | | | | 6.64 ± 2.37 | | | | | 4.13 ± 0.81 | | |
| Ratio of an A(H5N1) subtype $MedIC_{50}$ to an $IC_{50}$ of A/California/07/2009[c] | | | | 4.33 | | | | | 1.45 | | | | | 1.37 | | |
| Ratio of a B/Austria/1359417/2021 $IC_{50}$ to an A(H5N1) subtype $MedIC_{50}$ | | | | 18.21 | | | | | 13.55 | | | | | 26.20 | | |
| Ratio of an A(H5N5) subtype $MedIC_{50}$ to an $IC_{50}$ of A/California/07/2009 | | | | 1.63 | | | | | 3.37 | | | | | 1.02 | | |
| Ratio of a B/Austria/1359417/2021 $IC_{50}$ to an A(H5N5) subtype $MedIC_{50}$ | | | | 48.28 | | | | | 5.85 | | | | | 35.26 | | |
| Ratio of an X1 group $MedIC_{50}$ to an X2 group $MedIC_{50}$ | | | | 1.08 | | | | | 0.83 | | | | | 1.13 | | |

[a] - N - number of tested strains from an indicated group

[b] - descriptive statistics of the group: min, max - minimum and maximum $IC_{50}$ values in the group; Q1, Q3 - first and third quartiles.

[c] - $MedIC_{50}$ - median $IC_{50}$ determined for a group of viruses.

**Table S5. Pairwise comparison of median $IC_{50}$ values of oseltamivir, zanamivir and peramivir determined for various subtypes and NA genetic groups.**

| OSELTAMIVIR | P-values determined in post-hoc Dunn's test | | | | | | Ratio of a Group1 $MedIC_{50}$ to a Group2 $MedIC_{50}$ | | | | | |
|---|---|---|---|---|---|---|---|---|---|---|---|---|
| Group1↓/Group2→ | ea3.1 | ea3.2 | ea5 | ea5.1 | X2 | A(H5N5) | ea3.1 | ea3.2 | ea5 | ea5.1 | X2 | A(H5N5) |
| ea3.1 | | 0.10945 | 1 | 3.31E-05 | 1 | 0.12035 | | 0.85 | 1.00 | 30.58 | 1.09 | 2.68 |
| ea3.2 | | | 0.01182 | 1.13E-15 | 0.32854 | 3.58E-06 | | | 1.17 | 35.83 | 1.27 | 3.14 |
| ea5 | | | | 1.47E-09 | 1 | 0.01739 | | | | 30.71 | 1.09 | 2.69 |
| ea5.1 | | | | | 0.25982 | 1 | | | | | 0.04 | 0.09 |
| X2 | | | | | | 1 | | | | | | 2.46 |
| A(H5N5) | | | | | | | | | | | | |

| ZANAMIVIR | P-values determined in post-hoc Dunn's test | | | | | | Ratio of a Group1 $MedIC_{50}$ to a Group2 $MedIC_{50}$ | | | | | |
|---|---|---|---|---|---|---|---|---|---|---|---|---|
| Group1↓/Group2→ | ea3.1 | ea3.2 | ea5 | ea5.1 | X2 | A(H5N5) | ea3.1 | ea3.2 | ea5 | ea5.1 | X2 | A(H5N5) |
| ea3.1 | | 6.85E-09 | 0.82021 | 3.69E-07 | 0.24014 | 1.12E-09 | | 0.47 | 0.85 | 0.53 | 0.62 | 0.32 |
| ea3.2 | | | 3.15E-08 | 1 | 1 | 1 | | | 1.81 | 1.12 | 1.31 | 0.68 |
| ea5 | | | | 4.17E-06 | 1 | 2.15E-08 | | | | 0.62 | 0.73 | 0.38 |
| ea5.1 | | | | | 1 | 0.40419 | | | | | 1.17 | 0.60 |
| X2 | | | | | | 0.27659 | | | | | | 0.52 |
| A(H5N5) | | | | | | | | | | | | |

| PERAMIVIR | P-values determined in post-hoc Dunn's test | | | | | | Ratio of a Group1 MedIC50 to a Group2 MedIC50 | | | | | |
|---|---|---|---|---|---|---|---|---|---|---|---|---|
| Group1↓/Group2→ | ea3.1 | ea3.2 | ea5 | ea5.1 | X2 | A(H5N5) | ea3.1 | ea3.2 | ea5 | ea5.1 | X2 | A(H5N5) |
| ea3.1 | | 1 | 1 | 8.02E-04 | 0.50597 | 2.43E-05 | | 1.02 | 0.97 | 1.19 | 1.18 | 1.42 |
| ea3.2 | | | 1 | 0.00883 | 1 | 2.47E-04 | | | 0.95 | 1.16 | 1.15 | 1.38 |
| ea5 | | | | 9.20E-06 | 0.50209 | 1.16E-06 | | | | 1.23 | 1.22 | 1.46 |
| ea5.1 | | | | | 1 | 1 | | | | | 0.99 | 1.19 |
| X2 | | | | | | 1 | | | | | | 1.20 |
| A(H5N5) | | | | | | | | | | | | |
| $p \leq 0.05$ | $p \leq 0.01$ | $p \leq 0.001$ | $p \leq 0.0001$ | | | | | | | | | |

**Table S6. Influenza A(H5N1) and A(H5N5) viruses identified as outliers in a box-and whiskers plot analysis of oseltamivir, zanamivir and peramivir $IC_{50}$ values.**

| № | Outlier strain | Sub-type | NA clade | Oseltamivir | | | Zanamivir | | | Peramivir | | | Amino acid substitutions in NA compared to other representatives of a corresponding NA genetic group |
|---|---|---|---|---|---|---|---|---|---|---|---|---|---|
| | | | | Mean $IC_{50}$ ± SD, nM | fold change compared to | | Mean $IC_{50}$ ± SD, nM | fold change compared to | | Mean $IC_{50}$ ± SD, nM | fold change compared to | | |
| | | | | | subtype median | NA clade median | | subtype median | NA clade median | | subtype median | NA clade median | |
| 1 | A/chicken/Magadan/14-1 V/2022 | H5N1 | ea3.2 | 3.08 ± 0.069 | 1.78 | 1.50 | 1.35 ± 0.172 | 2.75 | 1.75 | 0.2 ± 0.001 | 1.30 | 1.26 | G41E, Q43R, T55A, A79T, V116I |
| 2 | A/chicken/Kurgan/72-1V/2021 | H5N1 | ea5 | 2.01 ± 0.371 | 1.16 | 1.14 | 0.68 ± 0.1 | 1.39 | 1.60 | 0.23 ± 0.036 | 1.48 | 1.36 | E47G, V83L, N209D, I467V |
| 3 | A/chicken/Orenburg/46-1V/2021 | H5N1 | ea5 | 1.41 ± 0.026 | 0.81 | 0.80 | 0.58 ± 0.11 | 1.17 | 1.35 | 0.15 ± 0.006 | 0.93 | 0.86 | K150N |
| 4 | A/chicken/Rostov-on-Don/159-1V/2021 | H5N1 | ea5 | 1.73 ± 0.102 | 1.00 | 0.99 | 0.6 ± 0.056 | 1.22 | 1.40 | 0.15 ± 0.002 | 0.95 | 0.88 | P46L, V116I, S386I |
| 5 | A/chicken/Rostov-on-Don/159-2V/2021 | H5N1 | ea5 | 1.81 ± 0.133 | 1.04 | 1.03 | 0.6 ± 0.013 | 1.23 | 1.41 | 0.14 ± 0.003 | 0.86 | 0.80 | P46L, V116I, S386I |
| 6 | A/chicken/Rostov-on-Don/159-3V/2021 | H5N1 | ea5 | 1.76 ± 0.126 | 1.01 | 1.00 | 0.6 ± 0.044 | 1.22 | 1.40 | 0.17 ± 0.013 | 1.11 | 1.02 | P46L, V116I, S386I |
| 7 | A/chicken/Rostov-on-Don/159-5V/2021 | H5N1 | ea5 | 1.77 ± 0.078 | 1.02 | 1.01 | 0.61 ± 0.01 | 1.24 | 1.43 | 0.15 ± 0.008 | 0.93 | 0.86 | P46L, V116I, S386I |
| 8 | A/goose/Chelyabinsk/34-1V/2021 | H5N1 | ea5 | 0.5 ± 0.018 | 0.29 | 0.29 | 0.35 ± 0.009 | 0.71 | 0.81 | 0.24 ± 0.027 | 1.52 | 1.40 | S442I |
| 9 | A/goose/Tyumen/359-12/2021 | H5N1 | ea5 | 1.06 ± 0.1 | 0.61 | 0.60 | 0.49 ± 0.004 | 1.00 | 1.15 | 0.31 ± 0.042 | 1.94 | 1.79 | E47G, V149D, I436V |
| 10 | A/chicken/Chelyabinsk/241-5V/2022 | H5N1 | ea5.1 | 0.06 ± 0.004 | 0.04 | 1.09 | 0.66 ± 0.04 | 1.34 | 0.96 | 0.16 ± 0.011 | 1.01 | 1.14 | V34I |
| 11 | A/chicken/Ryazan/228-3V/2022 | H5N1 | ea5.1 | 0.05 ± 0.005 | 0.03 | 0.85 | 0.7 ± 0.021 | 1.43 | 1.03 | 0.12 ± 0.004 | 0.75 | 0.85 | none |
| 12 | A/dalmatian pelican/Astraknan/344-12/2021 | H5N5 | ea6 | 1.15 ± 0.001 | 1.77 | 1.77 | 4.39 ± 0.308 | 3.87 | 3.87 | 0.13 ± 0.007 | 1.12 | 1.12 | D303N, I428V |
| Median $IC_{50}$, nM | A(H5N1) subtype | | | 1.73 | | | 0.49 | | | 0.16 | | | |
| | ea3.2 NA clade | | | 2.05 | | | 0.77 | | | 0.16 | | | |
| | ea5 NA clade | | | 1.76 | | | 0.43 | | | 0.17 | | | |
| | ea5.1 NA clade | | | 0.06 | | | 0.68 | | | 0.14 | | | |
| | A(H5N5) subtype | | | 0.65 | | | 1.13 | | | 0.12 | | | |

- upper outlier in a corresponding NA clade

- lower outlier in a corresponding NA clade

**Table S7. Analysis of NA sequences of influenza A(H5N1) 2.3.4.4b viruses deposited in the GISAID database for amino acid substitutions at the 253 position (excluding ea5.1 viruses detected in 2021-2022 in Russia described in this article).**

| Year of sample collection | Number of amino acid NA sequences deposited to GISAID analyzed | Amino acid residue at position 253 in NA | | |
|---|---|---|---|---|
| | | Y | H | S |
| 2020 | 20 | 20 | 0 | 0 |
| 2021 | 1307 | 1306 | 1 | 0 |
| 2022 | 9965 | 9951 | 11 | 3 |
| 2023 | 5428 | 5425 | 3 | 0 |
| 2024 | 7185 | 7180 | 4 | 1 |
| 2025 | 10638 | 10632 | 6 | 0 |
| **Total number** | **34543** | **34514** | **25** | **4** |
| *%* | | *99.92* | *0.07* | *0.01* |

**Parameters used for the database search:**

- A(H5N1) subtype;
- 2.3.4.4b HA clade;
- Availability of complete NA sequences;
- Collection dates from January 1 to December 31 of the indicated year;
- GISAID accession date August 19, 2026.

**Table S8. Influenza A(H5N1) 2.3.4.4b viruses bearing an NA-Y253H/S amino acid substitutions identified in the GISAID database (2020-2025, globally) (excluding ea5.1 viruses detected in 2021-2022 in Russia described in this article).**

| | **NA sequence (**Strain name\|GISAID ID\|Subtype\|Passage history\|HA clade\|Collection date\|Segment) | **Year of sample collection** | **Amino acid residue at position 253 in neuraminidase** |
|---|---|---|---|
| 1 | A/feces/Somalia/ID_146/2021\|EPI_ISL_19613471\|A/H5N1\|2.3.4.4b\|2021-01-01 | 2021 | H |
| 2 | A/feces/Sri_Lanka/ID_172/2022\|EPI_ISL_19613512\|A/H5N1\|2.3.4.4b\|2021-01-01 | 2022 | H |
| 3 | A/american_white_pelican/California/22-035599-001/2022\|EPI_ISL_19606689\|A/H5N1\|2.3.4.4b\|2022-10-31 | 2022 | H |
| 4 | A/Canada_Goose/AB/FAV-0505-15/2022\|EPI_ISL_19157540\|A/H5N1\|2.3.4.4b\|2022-04-26 | 2022 | H |
| 5 | A/canada_goose/BC/AIVPHL-186/2022\|EPI_ISL_17051372\|A/H5N1\|2.3.4.4b\|2022-06-22 | 2022 | H |
| 6 | A/Canada_Goose/BC/FAV-0899-1/2022\|EPI_ISL_19152710\|A/H5N1\|\|2.3.4.4b\|2022-06-02 | 2022 | H |
| 7 | A/chicken/Korea/H633/2022\|EPI_ISL_19453465\|A/H5N1\|2.3.4.4b\|2022-11-28 | 2022 | H |
| 8 | A/duck/Vietnam/NCVD-22AS469-11/2022\|EPI_ISL_19866158\|A/H5N1\|2.3.4.4b\|2022-07-29 | 2022 | H |
| 9 | A/great_horned_owl/Minnesota/22-014521-001/2022\|EPI_ISL_19607244\|A/H5N1\|2.3.4.4b\|2022-05-05 | 2022 | H |
| 10 | A/great_horned_owl/Minnesota/22-014521-002/2022\|EPI_ISL_19607245\|A/H5N1\|2.3.4.4b\|2022-05-05 | 2022 | H |
| 11 | A/horned_grebe/California/22-037519-002/2022\|EPI_ISL_19607626\|A/H5N1\|\|2.3.4.4b\|2022-11-04 | 2022 | H |
| 12 | A/Mixed/AB/FAV-1367-3/2022\|EPI_ISL_19154672\|A/H5N1\|\|2.3.4.4b\|2022-11-01 | 2022 | H |
| 13 | A/chicken/Belgorod/283-1V/2023\|EPI_ISL_18462486\|A/H5N1\|2.3.4.4b\|2023-01-26 | 2023 | H |
| 14 | A/chicken/New_York/NYCVH-168127/2023\|EPI_ISL_19101498\|A/H5N1\|2.3.4.4b\|2023-04-09 | 2023 | H |
| 15 | A/chicken/Scotland/006955/2023\|EPI_ISL_16811191\|A/H5N1\|2.3.4.4b\|2023-01-11 | 2023 | H |
| 16 | A/turkey/Missouri/038169-001/2024\|EPI_ISL_19736973\|A/H5N1\|2.3.4.4b\|2024-12-19 | 2024 | H |
| 17 | A/turkey/Missouri/038169-002/2024\|EPI_ISL_19736972\|A/H5N1\|\|2.3.4.4b\|2024-12-19 | 2024 | H |
| 18 | A/turkey/Missouri/038618-001/2024\|EPI_ISL_19737001\|A/H5N1\|2.3.4.4b\|2024-12-23 | 2024 | H |
| 19 | A/turkey/Missouri/038618-002/2024\|EPI_ISL_19737000\|A/H5N1\|2.3.4.4b\|2024-12-23 | 2024 | H |
| 20 | A/canada_goose/USA/001439-001/2025\|EPI_ISL_19870506\|A/H5N1\|2.3.4.4b\|2025-01-01 | 2025 | H |
| 21 | A/Mute_Swan/England/387795/2025\|EPI_ISL_20298268\|A/H5N1\|2.3.4.4b\|2025-10-24 | 2025 | H |
| 22 | A/Mute_Swan/England/429423/2025\|EPI_ISL_20298471\|A/H5N1\|2.3.4.4b\|2025-10-26 | 2025 | H |
| 23 | A/great_horned_owl/Colorado/06819-001/2025\|EPI_ISL_20378010\|A/H5N1\|2.3.4.4b\|2025-12-23 | 2025 | H |
| 24 | A/american_wigeon/Oregon/04573-001/2025\|EPI_ISL_20329806\|A/H5N1\|2.3.4.4b\|2025-11-19 | 2025 | H |
| 25 | A/common_eider/Massachusetts/25WI00081/2025\|EPI_ISL_20419086\|A/H5N1\|2.3.4.4b\|2025-04-25 | 2025 | H |
| 26 | A/chicken/Choco/ICA-3502/2022\|EPI_ISL_17353508\|A/H5N1\|2.3.4.4b\|2022-11-13 | 2022 | S |
| 27 | A/chicken/Choco/ICA-3504/2022\|EPI_ISL_17353509\|A/H5N1\|2.3.4.4b\|2022-11-01 | 2022 | S |
| 28 | A/duck/Choco/ICA-3501/2022\|EPI_ISL_17353839\|A/H5N1\|2.3.4.4b\|2022-10-09 | 2022 | S |
| 29 | A/Red-Tailed_Hawk/ON/FAV-0081-1/2024\|EPI_ISL_19776246\|A/H5N1\|2.3.4.4b\|2024-02-18 | 2024 | S |
