# Supplementary material for: Antiviral Susceptibility of Clade 2.3.4.4b H5N1 and H5N5 Influenza Viruses Detected in Birds in Russia During 2021–2022
Source: Viruses. 2026 Oct 10;18(10):1126. doi: 10.3390/v18101126 (PMC13709846; doi:10.3390/v18101126)
Supplement: Supplementary file 1 [file viruses-18-01126-s001.zip › Table S10.pdf]

Supplementary Material

All genome sequences and associated metadata supporting the findings of this study can be accessed through the persistent digital object identifier https://doi.org/10.55876/gis8.260821nb

In addition to the minted DOI, GISAID also communicates the aggregation of GISAID accession numbers (EPI_ISL_IDs) through the corresponding EPI_SET_260821nb identifier to facilitate both, the acknowledgment of all data contributors and the direct retrieval of the underlying data from GISAID used in this study.

Table S10. Influenza Virus Data Summary

GISAID Identifier	Digital Object Identifier	Number of individual viruses	Data Collection range	Number of countries/territories
EPI_SET_260821nb	https://doi.org/10.55876/gis8.260821nb	173	1999-10-07 to 2025-12-23	42
